# Graphene-Loaded Aphron Microbubbles for Enhanced Drilling Fluid Performance and Carbon Capture and Storage

**DOI:** 10.1021/acsanm.4c05693

**Published:** 2024-11-11

**Authors:** Mohammad Hossein Akhlaghi, Malek Naderi, Mojtaba Abdi-Jalebi

**Affiliations:** † Department of Materials and Metallurgical Engineering, Amirkabir University of Technology (Tehran Polytechnic), 1591634311 Tehran, Iran; ‡ Graphene and Advanced Materials Laboratory (GAMLab), Innovation tower of Amirkabir University of Technology (Tehran Polytechnic), 1591634311 Tehran, Iran; § Institute for Materials Discovery, University College London, Malet Place, London WC1E 7JE, U.K.

**Keywords:** aphron microbubble, CO_2_ storage, water-based drilling fluid, nanosheet, partially reduced graphene oxide, stability

## Abstract

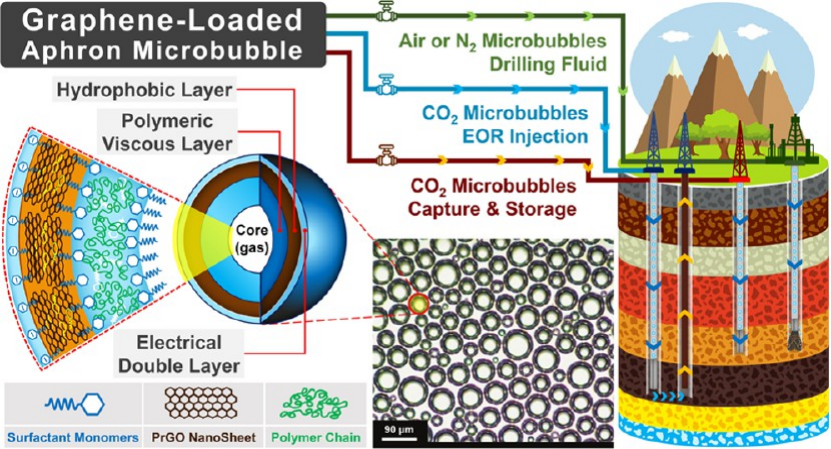

Maintaining the stability of microbubbles is essential for enhancing the longevity of aphronic water-based drilling fluid usage in drilling depleted reservoirs and other under-pressured zones. Here, we introduce the integration of partially reduced graphene oxide (PrGO) nanosheets into the shell of aphron microbubbles (AMBS) to enhance the stability and size distribution, particularly for drilling fluid applications and carbon geological storage. The amphiphilic characteristic of PrGO nanosheets, due to meticulous control of the reduction process of graphene oxide, facilitates their spontaneous adsorption at interfaces, thereby reducing the interfacial energy as a two-dimensional surfactant. The loading of PrGO nanosheets in the polymeric shell of AMBs enhances mechanical strength, stability, and resistance to gas diffusion, prolonging the half-life of the microbubbles to over 120%. According to the results, a more uniform size distribution and reduction of microbubble size up to 60% have been achieved at a concentration of 0.30 wt % PrGO. Rheological studies using various models indicate the optimal PrGO concentration for improved stability in aphronic fluids. Filtration tests indicate the loaded PrGO can reduce filtration loss by up to 45% at 0.50 wt % by forming a cohesive and compressible cake, improving filtration control. Changes in the Fourier-transform infrared spectra and contact angle measurements suggest increased surface hydrophobicity with higher graphene concentrations in aphronic fluid cakes. Moreover, the study elucidates that the stability of microbubbles is influenced by the type of core gas, with N_2_ gas yielding the highest stability and CO_2_ the lowest. Ultimately, these results highlight the beneficial effect of incorporation of PrGO nanosheets in aphron microbubbles to boost the drilling fluid performance and efficiency, which will pave the way toward ultralightweight fluids that can even be used as carrier and injection fluids in carbon capture and leak-free geological storage technologies.

## 1 Introduction

One of the significant challenges currently confronting the drilling industry is the process of drilling in low-pressure or depleted reservoirs. This particular type of drilling presents a myriad of technical and economic obstacles, such as unmanageable loss in fractures and pipe sticking, ultimately rendering the development of these fields economically unsustainable.^1^

Recent advancements in the drilling industry have led to the development of lightweight and ultralightweight fluids aimed at improving the efficiency of drilling, well completions, and workover operations. These innovative fluids, utilizing aphron colloidal microbubbles, have been found to effectively meet the needs of drilling and workover operations while also reducing damage to the formation.^1−3^ The design and formulation of aphronic microbubble fluid technology emphasize the control of fluid flow into the formation to prevent overflow and minimize formation damage.^4^ Producing drilling fluid with lower density and enhanced pore-blocking properties based on pressure differentials is a fundamental aspect of deploying this advanced technology.^5^

Aphron microbubbles are characterized by their colloidal stability within a liquid continuous phase, displaying a unique core–shell structure. The special properties exhibited by these microbubbles are dependent on the phases present within the core, shell, and continuous phase. Aphron microbubbles (AMBS) have unique properties, including large specific surface area and controllable surface properties, relatively great stability, high compressibility, easy separation from the bulk medium, and flow properties close to those of water, which make them proper candidates for different applications, such as biotechnology^6−8^ and drug delivery,^9−11^ smart-fluid technology,^3,12^ separation industry,^13^ protein recovery and flotation process,^14^ oil and gas drilling fluids,^15−18^ CO_2_-EOR and CO_2_ geological storage in carbon capture and storage (CCS) technologies,^19^ and materials synthesis.^20^

The composition of the shell, consisting of a two- or three-layer structure of surfactant molecules, and the nonuniform size distribution of the microbubbles due to the high permeability of the gaseous core to the continuous phase contribute to the lack of stability in practical conditions.^3^

Based on the unique properties of aphrons and the combination of colloidal gas aphrons with water-based drilling fluid in order to create microbubble drilling fluid, many research studies have been conducted, which show high application potential and strong interest in its field application and operational optimization as an advanced technology in the oil and gas industry today.^2,21,22^ However, a major challenge faced by these fluids is their stability at varying drilling depths. The stability of microbubbles and the changes in bubble size under different drilling conditions have been identified as key issues hindering the widespread application of aphron-based drilling fluids.^23^ Achieving optimal efficiency and maximum utilization in the design of a functional fluid requires stable aphrons in smaller dimensions with enhanced performance through increased surface energy. Smaller aphrons lead to a greater interfacial area and active surface, resulting in improved stability and efficiency in practical applications. Consequently, attaining a narrower size distribution and a smaller size with enhanced stability of aphrons is one of the other problems and challenges of this type of fluid.^17^

Microbubble-based drilling fluids exhibit instability during drilling, leading to a significant drop in performance after multiple circulation cycles in the well.^24^ Despite the advantages offered by this technology, its limited stability poses a barrier to widespread adoption. Addressing the stabilization of properties and performance of aphron-based fluids is crucial in overcoming these challenges.

In recent years, efforts have been focused on enhancing the stability of aphron-based drilling fluids under varying temperature and pressure conditions through the implementation of different methods and the use of novel or improved additives.^17,18,25−28^

Aphron microbubbles are complex dynamic systems characterized by continual changes in their structure resulting from bubble breakup, coalescence, and fission, leading to limited stability. Various parameters have been established for these microbubbles by researchers.^3,29^ The primary parameter determining the quality of aphron microbubbles is their stability, which surpasses that of foams and pseudofoams. Stability is influenced by factors such as shell thickness, shell viscosity, and the type and concentration of surfactant and polymer. The stability of aphron microbubbles is critical for their practical applications owing to their thermodynamic quasi-stable nature. Despite their superior stability compared to traditional foams, aphron microbubbles can undergo shape changes over time due to transfer and penetration processes, transitioning from a spherical state to a more complex, multifaceted shape.^30^

Efforts to enhance the stability and longevity of thermodynamically unstable microbubbles have focused on introducing coatings or surface layers of lipids,^31^ proteins,^32^ surfactants,^33^ polymers,^34^ nanoparticles,^35,36^ or combinations thereof. Selecting appropriate shell materials is critical for mechanical properties and stability, with potential strategies including incorporating solid particles or suitable fillers in the polymer matrix to reinforce the shell and reduce permeability.^37,38^ In recent years, graphene-based nanostructures have emerged as promising options for enhancing the mechanical properties of polymer matrices due to their unique characteristics.^39,40^ Graphene nanosheets are employed as high-performance fillers in polymer nanocomposites to enhance both mechanical and physical attributes.^41^ Graphene has been increasingly recognized for its remarkable applications, including its use as an active component in water-based drilling fluids. The incorporation of graphene into these fluids offers a range of benefits, such as enhanced thermal stability, minimized fluid loss, and superior lubricating properties. These advantages can significantly improve the effectiveness and efficiency of drilling operations in the oil and gas industry.^42,43^

To enhance efficiency and utilize practical design effectively, incorporating graphene-based nanostructures into the shell of aphron microbubbles can significantly improve the stability and size distribution. Through the lens of interfacial science, precise control over the oxygen functionality during the reduction process of graphene oxide can result in tailored surface energies and hydrophobicity, thereby yielding unique and appealing interfacial properties.^44,45^ By carefully modulating the rate of reduction of graphene oxide, materials with versatile surfaces can be synthesized, exhibiting distinctive properties and applications. Following partial reduction, the graphene sheets undergo derivatization to form a two-dimensional lattice composed of partially fragmented sp^2^-bonded carbon networks bearing phenol, hydroxyl, and epoxide groups on the basal planes, as well as carboxylic acid groups at the edges.^44^ This structure renders partially reduced graphene oxide (PrGO) amphiphilic with a sizable hydrophobic basal plane and hydrophilic edges. Owing to the distribution of both hydrophilic and hydrophobic groups on PrGO nanosheets, it can spontaneously adsorb to interfaces and reduce interfacial energy, serving as a two-dimensional surfactant.^46^

In this research, we have provided a pioneering approach to deal with the challenges related to the stability and uniformity of size distribution in the technology of aphronic water-based drilling fluids by embedding and integrating PrGO nanosheets in the shells of aphron microbubbles. The extraordinary properties of graphene structure significantly contribute to increasing the stability of aphron microbubbles, preventing coalescence and the Ostwald ripening phenomenon. This innovative advancement enables precise applications in lightweight and ultralightweight water-based drilling fluids, with ongoing research offering the potential to enhance current technologies and drive future innovations in CO_2_ geological storage.

## 2 Materials and Methods

### 2.1 Materials

Graphite powder −325 mesh (C, ≥98.5%), sodium nitrate (NaNO_3_, ≥99.0%), hydrogen peroxide (H_2_O_2_, 35 wt % in H_2_O), sodium dodecyl benzenesulfonate (SDBS, C_18_H_29_NaO_3_S, ≥98.5%), polysorbate 20 (T20, C_58_H_114_O_26_, ≥40.0%), and Xanthan gum polymer (XG, C_8_H_14_Cl_2_N_2_O_2_, ≥90.0%) were purchased from Sigma-Aldrich. Sulfuric acid (H_2_SO_4_, 98%), hydrochloric acid (HCl, 37.0 wt %), potassium permanganate (KMnO_4_, ≥99.0%), sodium carbonate (Na_2_CO_3_, ≥99.5%), sodium hydroxide (NaOH, ≥99.0%), and ethyl alcohol (EtOH, C_2_H_6_O, ≥99.0%) were supplied by Merck Company. Zwitterionic surfactant cocamidopropyl betaine (CPB, C_19_H_38_N_2_O_3_, ≥30.0%) was provided by DChemie chemical supplies. Low-viscosity polyanionic cellulose (PAC-LV), modified starch, and biocide were purchased from M-I SWACO supplies as technical-grade drilling fluid additives. The gases used for the experiments were 99.9% pure CO_2_ and 99.6% pure N_2_.

All of the chemical reagents were used as received without further purification. All aqueous solutions were prepared with deionized water (DW).

### 2.2 Preparation of PrGO Nanosheets

GO sheets were synthesized via the ultrasonic-assisted modified Hummer’s method.^47^ Briefly, graphite powder (3 g, 250 mmol) was added to a concentrated H_2_SO_4_ and H_3_PO_4_ mixed solution (9:1 volume ratio). Then, KMnO_4_ (18 g, 114 mmol) was slowly added to the mixture and magnetically stirred for 5 min. After that, the mixture was sonicated for 45 min at 35 °C with an ultrasonic bath sonicator (SONICA 2400 S3, SOLTEC Srl, 40 kHz, 260 W), and a dark brown mixture was obtained. The reaction mixture was cooled and poured onto ice of DW (400 mL) with 30% H_2_O_2_ (3 mL). The resultant yellow suspension was subsequently filtered and washed with diluted HCl solution (10%), followed by rewashing with DW and ETOH centrifugation several times at 4000 rpm. Finally, the prepared GO was dried at 40 °C in a vacuum oven to decrease the amount of trapped water in the GO structure. For preparing PrGO, the dried GO powder was reduced thermally at 300 °C for 5 min in a box furnace.

### 2.3 Preparation of the Aphronic Water-Based Drilling Fluid

**Scheme 1:**
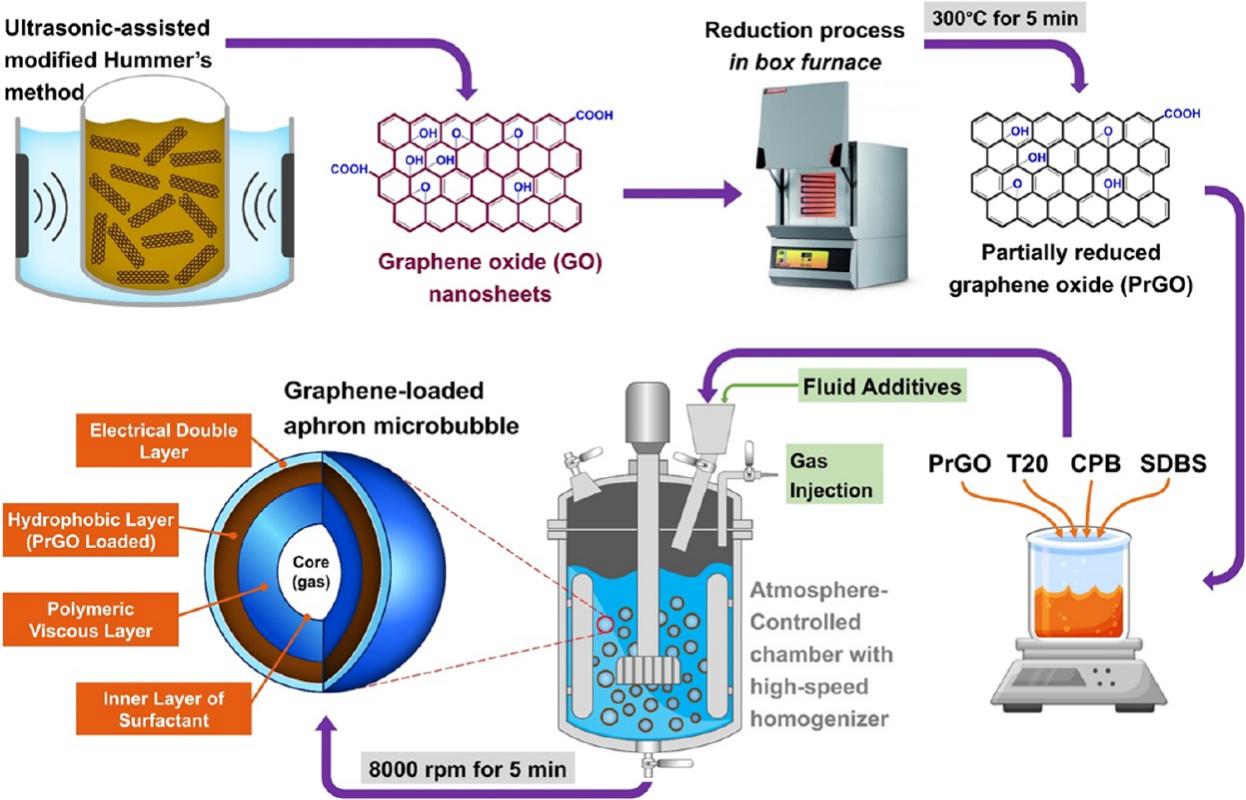
Schematic Illustration of the Synthesis Route: Schematic Representation Showing the Method Used for Synthesizing Graphene-Loaded Aphron Microbubbles, along with the Final Structure of the Product

The AMB water-based drilling fluids are formulated from eight components, as shown in Table S1, Supporting Information. The microbubble fluid was prepared using a high-speed homogenizer (IKA T-25, Cole-Parmer Germany) equipped with a chamber capable of maintaining a gas atmosphere of air, N_2_, or CO_2_. Components were added based on specified concentrations and mixed for designated times. At this stage, care should be taken not to form a foam. Subsequently, the desired gas was introduced into the chamber and mixed at 8000 rpm for 5 min. Shearing speed and time were standardized to ensure the consistent creation of microbubbles across all samples. Prior to introducing N_2_ or CO_2_ gases, the system was evacuated to prevent air contamination during microbubble production. In order to prepare the mixed surfactant solution, three types of anionic (SDBS) as a base active agent, nonionic (T20) as a stabilizing agent, and zwitterionic (CPB) as a booster agent were used. In this regard, a mixed aqueous solution (7.5 mL) containing SDBS (0.05 g, 0.14 mmol), T20 (0.25 g, 0.20 mmol), and CPB (0.10 g, 0.30 mmol) was prepared at room temperature. To investigate the effect of PrGO concentration, six concentrations of 0.05 wt % (GAMB-05), 0.10 wt % (GAMB-10), 0.20 wt % (GAMB-20), 0.30 wt % (GAMB-30), 0.40 wt % (GAMB-40), and 0.50 wt % (GAMB-50) were considered and in the surfactant solution added and mixed. To compare with the base case, a blank AMB without PrGO was also prepared according to the same process and conditions. Scheme 1 provides a schematic representation of the synthesis route for the graphene-loaded aphron microbubble. This scheme depicts both the process of synthesis and the structure of the resulting graphene-loaded aphron microbubble.

### 2.4 Characterization

The morphological features of the obtained PrGO nanosheets were studied by field emission scanning electron microscopy (FE-SEM) (MIRA3-XMU Tescan) and transmission electron microscopy (TEM) on a Philips CM120 with an accelerating voltage of 100 kV. X-ray diffraction (XRD) patterns were recorded on a Philips PW 1730 X-ray diffractometer with Cu Kα radiation (λ = 1.5418 Å) operated at 40 kV and 30 mA in the 2θ range of 5–60° at a scanning step of 0.05°. The functional groups and chemical bonds were evaluated by a Spectrum 400 HR Fourier transform infrared spectroscopy system (FTIR). The carbonic structure of synthesized GO and PrGO was investigated by Raman spectroscopy (TakRam N1–541, TeKsan). The average diameter and size distribution of AMBs were determined using a transmission optical microscope (BM-500, Sairan) and were analyzed using ImageJ software. The drilling fluid properties were tested in accordance with the standards set by the American Petroleum Institute (API). Rheological properties of AMB drilling fluids were analyzed using a six-speed viscometer (Model 35SA, Fann) and a low-speed viscometer (Model DV2T, Brookfield). Filtration testing was conducted utilizing an API multiple-unit filter press apparatus (LPLT, Fann) under a pressure of 100 psi, with the volume of fluid loss recorded over a 30 min period.

## 3 Results and Discussion

### 3.1 Structural Characterization of Partially Reduced Graphene Oxide

**Figure 1:**
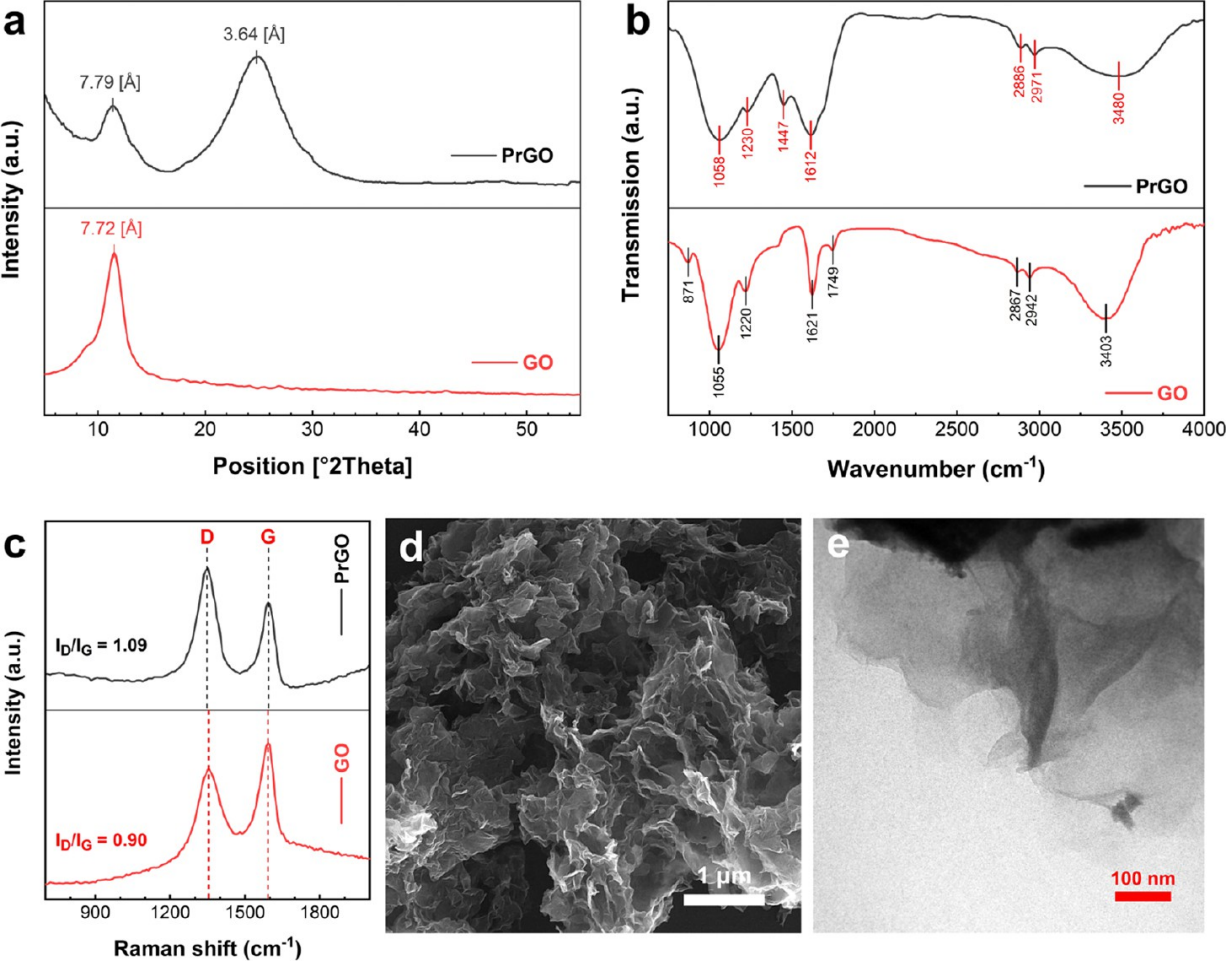
Characterization results of the as-synthesized PrGO nanosheets. (a) XRD: decreased *d*-spacing of graphene sheets due to thermal reduction and removal of a part of functional groups, (b) FTIR: decrease in oxygenated functional groups in PrGO compared to GO, (c) Raman: confirmation of PrGO formation with increased sp^3^ defects and decreased sp^2^ domains, (d) FE-SEM, and (e) TEM: two-dimensional structure of PrGO with crumpled and wrinkled graphene nanosheets.

Spectroscopy and electron microscopy results of the as-synthesized GO and PrGO nanosheets are listed in Figure 1. As shown in Figure 1a, a sharp diffractive peak at about 2θ = 11.5° can be seen in the spectrum of GO, which corresponds to the (001) diffractive plane with a *d*-spacing of 7.72 Å. The large interlayer distance is attributed to the formation of hydroxyl, epoxy, and carboxyl groups.^48^ In contrast, this peak intensity has decreased in the spectrum of PrGO, and a broad peak is observed at around 2θ = 24.8°, which corresponds to the (002) plane with a *d*-spacing of 3.64 Å. As expected, after thermal reduction and partial removal of oxygen-containing functional groups, the π–π stacking between graphene sheets increased and caused the distance between the layers to decrease. From Figure 1b, the changes of the functional groups in the thermally reducing process were investigated by FTIR spectroscopy. The oxygenated functional groups such as C–O stretching vibration (carboxyl and alkoxy) at 1055 cm^–1^, C–O–C stretching (epoxy) at 1220 cm^–1^, C=C (aromatic) and O–H stretching and bending at 1621 cm^–1^, C=O stretching vibration (carbonyl) at 1749 cm^–1^, and O–H stretching (hydroxyl) at 3403 cm^–1^ appeared in GO spectra.^49^ As can be observed in the PrGO spectra, these characteristic groups of GO have become relatively weaker or have disappeared after thermal reduction. Therefore, it can be concluded that the deoxygenation leads to the PrGO becoming more hydrophobic, and the remaining water molecules are repelled from the interlayer pores. The formation of PrGO can be confirmed by Raman spectroscopy (Figure 1c). Two characteristic D and G bands were observed in both the GO and PrGO spectra. The D peak at around 1345–1355 cm^–1^ corresponds to structural defects and disorders in the graphite-like materials, whereas the G band at approximately 1590–1600 cm^–1^ is due to C–C stretching vibrations (sp^2^ carbons in rings and chains).^50^ The intensity ratio (*I*_D_/*I*_G_) that is attributed to sp^3^ hybridized carbon and sp^2^ carbon moieties increased from 0.90 to 1.09 after reduction. It can be ascertained that sp^3^ defects are increased in PrGO, and due to the partial removal of the oxygenated functional groups, sp^2^ domains are decreased during the reduction process. Consequently, it can be emphasized that the reducing reaction successfully proceeded. As can be seen in the electron microscopy (FE-SEM and TEM) images (Figure 1d,e), the PrGO structure has a two-dimensional structure containing crumpled and wrinkled graphene nanosheets.

### 3.2 Physical and Structural Characterizations of Aphron Microbubbles

Physical characterization of AMBs initially includes considerations of aphronic yield (amount of generated AMBs compared to the primary fluid), colloidal stability of microbubbles, and the amount of gas hold-up (volume percentage of entrapped gas in the microbubble core). The aphronic yield (*Y*_a_) and gas hold-up (GHU) percent can be obtained from eqs 1 and 2, respectively.
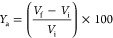
1
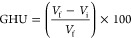
2where *V*_f_ and *V*_i_ are the volume of the fluid immediately after the formation of the AMBs and the volume of the initial fluid, respectively. The AMB stability, one of the most important parameters, is expressed in terms of when half the initial volume of liquid is drained (τ_1/2_, half-life time) that can be determined directly from the measurement of the volume of liquid drained with respect to time (drainage rate).

**Figure 2:**
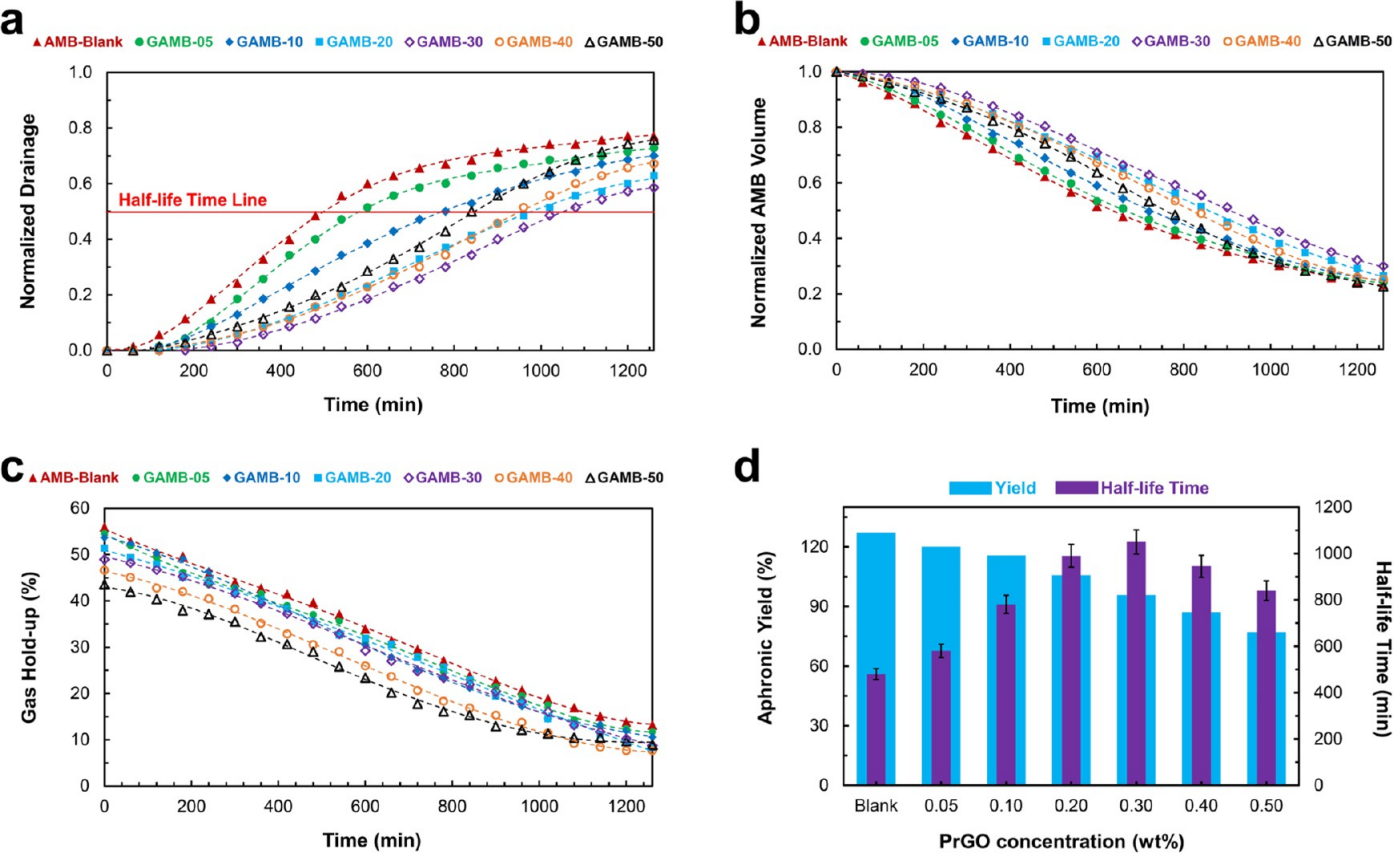
Physical characterization results of graphene-loaded aphron air-filled microbubbles. Time-behavior of (a) normalized drainage liquid, (b) normalized microbubble volume, (c) gas hold-up, and (d) yield and half-life time at various concentrations of PrGO. Experiments were carried out under atmospheric air pressure and constant temperature conditions of 26–28 °C.

The changes in aphronic parameters with time in the air-filled AMB water-based drilling fluids with different concentrations of PrGO are listed in Figure 2. As can be seen in Figure 2a, the amount of liquid drainage in the sample with a concentration of 0.30 wt % had a slower trend than other samples and started later and also ended later. In the same way, according to Figure 2b, it can be seen that the microbubbles produced in the concentration of 0.30 wt % of graphene have a slower change process with time and actually disappear later than other samples. Changes related to the amount of trapped air (GHU) with time for the prepared samples are listed in Figure 2c. The initial analysis shows that the blank sample initially contained a higher percentage of trapped air. However, with the introduction of PrGO, the percentage of trapped air decreased. As the concentration of PrGO increased, the amount of trapped air further decreased. This phenomenon may be attributed to the presence of a greater hydrophobic proportion of PrGO in the fluid, resulting in an antibubble behavior^51^ also also reducing the aphronic yield (Figure 2d).

The incorporation of PrGO nanosheets into the microbubble shell can increase the mechanical strength and stability of the shell structure due to the excellent mechanical properties of graphene and its amphiphilic behavior at the liquid–gas interface.^52^ The high surface area of PrGO nanosheets allows better interaction between the surfactant molecules and PrGO,^53^ leading to a more stable microbubble structure. In addition, PrGO nanosheets can act as a barrier to prevent gas diffusion from the microbubbles, leading to a longer half-life (stability) of the microbubbles. The high surface area of PrGO nanosheets provides a large contact surface to which surfactant molecules adhere, forming a stronger shell that can resist the diffusion of gas molecules. This barrier effect helps the microbubbles maintain their integrity and stability over a longer period of time.

**Figure 3:**
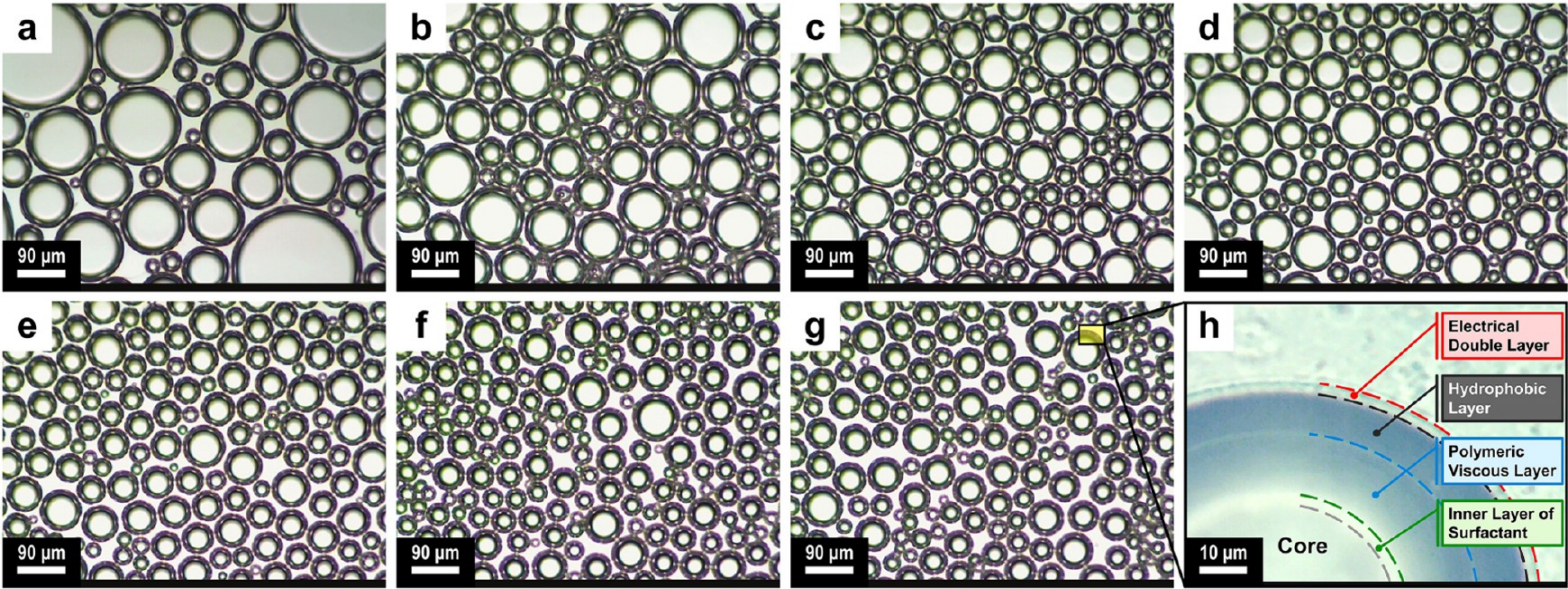
Optical micrographs of graphene-loaded aphron air-filled microbubbles. The effect of PrGO concentrations: (a) blank, (b) 0.05 wt %, (c) 0.10 wt %, (d) 0.20 wt %, (e) 0.30 wt %, (f) 0.40 wt %, and (g) 0.50 wt %. (h) three-layered structure of microbubble shell. The structural composition of aphron microbubbles bears resemblance to that of a core–shell structure, with multiple layers of surfactant molecules forming the shell. The distribution of surfactants within the structure is such that the hydrophobic or nonpolar heads are oriented toward the core, while the hydrophilic heads are positioned on the exterior. The shell comprises a dense three-layer film, consisting of an inner layer of surfactant surrounding the core, a viscous layer of continuous phase, and a two-layer structure of surfactant that enhances the strength and penetration resistance of the shell. The outer surface of aphrons may exhibit either a negative or positive charge, depending on the ionization of anionic or cationic surfactants. Consequently, the stability of the aphrons surpasses that of regular bubbles enveloped by a monolayer of surfactant molecules.

**Figure 4:**
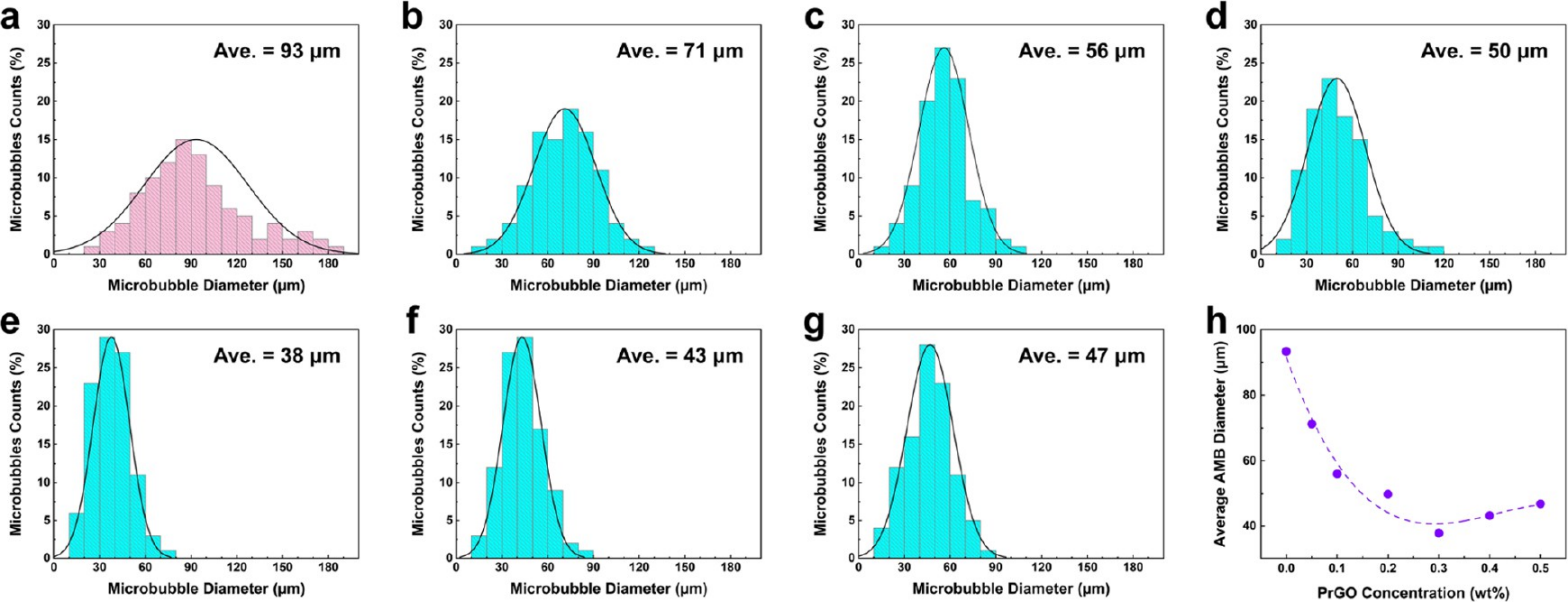
Microbubble size distribution of graphene-loaded aphron air-filled microbubbles. (a) blank, (b) 0.05 wt %, (c) 0.10 wt %, (d) 0.20 wt %, (e) 0.30 wt %, (f) 0.40 wt %, (g) 0.50 wt % PrGO, and (h) the effect of PrGO concentrations on average AMB diameter.

Optical microscopy images and size distribution of AMBs in different concentrations of PrGO are shown in Figures 3 and 4, respectively. According to the results, the microbubble size is reduced, and their size distribution is narrowed by strengthening bubble shells with PrGO. These changes in the microbubble size distribution indicate that the presence of PrGO nanosheets in the shell helps prevent drainage and air gas diffusion between the AMBs. By increasing the PrGO concentration up to 0.30 wt %, smaller microbubbles with a more uniform distribution were obtained, so the average size of microbubbles decreased from 71 μm for 0.05 wt % to 38 μm for 0.30 wt %.

However, with a further increase in the concentration, the mean shifts to a higher microbubble size. At low concentrations, the arrangement of graphene sheets in the shell is not complete, and the mechanisms of steric stabilization and surfactant transfer can be inhibited to some extent. By increasing the concentration to a critical value, the layer-by-layer density of graphene sheets in the shell becomes greater and can minimize free energy and stabilize microbubbles against collapse and drainage. At higher concentrations, the microbubble shell is saturated with PrGO nanosheets, and the rest remain in the continuous phase around the microbubbles.

Although graphene oxide itself also shows amphiphilic properties, due to its high negative surface charge, its absorption in the microbubble shell by itself is not feasible.^44^ Here, we were able to do this process by combining two modes; one is about the partial reduction process of graphene oxide, which can reduce its negative charge by eliminating some oxygen groups and enhancing its tendency to be absorbed at the interface.

The second mechanism focused on the interaction of graphene sheets with surfactants. Surfactants adhere to the surface of graphene sheets through electrostatic and steric forces or hydrophobic/hydrophilic interactions, augmenting their surface activity.^54^ This interaction enables the guided movement of graphene sheets to the microbubble shell with reduced energy expenditure, prompting a reorganization in the hydrophobic region of the shell. Consequently, the formation of a multilayered structure, facilitated by the arrangement of surfactant molecules and self-assembly of graphene sheets within the microbubble shell, significantly enhances the elasticity and mechanical strength of the shell, enabling the microbubbles to withstand external forces and maintain their shape over extended periods of time. Notably, the presence of graphene sheets establishes an effective gas barrier in the multilayered structure, limiting gas permeability and preventing bubble expansion, thereby achieving exceptional stability.

Previous studies have shown that the absorption of graphene sheets at the water–air or water–oil interface reduces surface tension.^55,56^ Therefore, the surface tension results can be used to prove and confirm the incorporation process of the PrGO nanosheets. The data (Figure S1a, Supporting Information) demonstrates a decline in surface tension up to a concentration of 0.20 wt % of PrGO with a steep slope, followed by a gradual decrease with increasing concentrations without a significant change in surface tension value. Further increases in PrGO concentration up to a critical threshold result in improved absorption within the microbubble shell, leading to thickening of the shell and prolonging the coalescence and drainage of the bubble, ultimately enhancing the microbubble stability. However, thermodynamically and based on the hydrodynamic properties of aphrons, there exists a finite maximum thickness for the shells of both stable and semistable microbubbles^3^ that limits the absorption of PrGO nanosheets in the hydrophobic layer after reaching the critical concentration. Consequently, excess PrGO remains in the surrounding medium. This surplus of PrGO at a fixed surfactant concentration can adsorb more surfactant molecules, ultimately diminishing their role in the microbubble formation process and thereby reducing the aphronic efficiency. Figure S1b in the Supporting Information depicts a detailed schematic illustrating the self-assembly process of shell layers during microbubble formation. This process involves the formation of a viscous polymeric layer, the interplay with the PrGO layer facilitated by the surfactants, and the development of an electrical double layer due to the negative charge of the anionic surfactant (SDBS). This visualization highlights the complex interactions and mechanisms that lead to the formation of graphene-loaded AMB.

To analyze the data collected from dispersion and determine the primary mechanism affecting the changes in drainage liquid quantity and microbubble volume, the Lifshitz–Slyozov–Wagner (LSW) theory can be utilized. This theory is associated with the Ostwald ripening phenomenon, describing the coarsening process of bubbles in a continuous phase.^57,58^ According to this phenomenon, the growth of the bubble size is linked to the consumption of smaller bubbles in the system, leading to their disintegration and eventual disappearance.

Within the framework of Ostwald ripening, scaling laws have been suggested for two distinct growth scenarios. In the diffusion-controlled ripening regime, the relationship between the radius of bubbles (*r*) and time (*t*) is described by a cubic relationship (*r*^3^ ∝ *t*). Conversely, in the interface-controlled ripening regime, the radius of bubbles scales with the square root of time. This implies that, in diffusion-controlled ripening, the bubbles grow larger with time at a faster rate compared to the interface-controlled regime, where the growth is slower.^59^ The incorporation of PrGO nanosheets into the microbubble shell can change the growth scenario. The presence of PrGO nanosheets in the shell at low concentrations can potentially reduce diffusion processes in the diffusion-controlled ripening regime and lead to lower growth rates. By increasing the concentration and placement of more graphene sheets in the shell, the interface between the bubble and the surrounding environment is disturbed, and surface tension effects become more important and can lead the ripening scenario to the interface-controlled ripening regime. As a result, this can lead to a square root relationship between bubble radius and time, the growth rate is better restrained, and higher stability is achieved.

### 3.3 Influence of Core Gas Type on Aphron Microbubbles Properties

The stability of the aphron microbubbles is significantly impacted by the type of core gas utilized to produce the bubbles. This core gas not only influences the average size and size distribution of the microbubbles but also plays a key role in their overall stability performance. In this section, we explored the impact of core gas type (air, N_2_, and CO_2_) on the stability performance, average size, and size distribution of aphron microbubbles reinforced with 0.30 wt % PrGO nanosheets.

**Figure 5:**
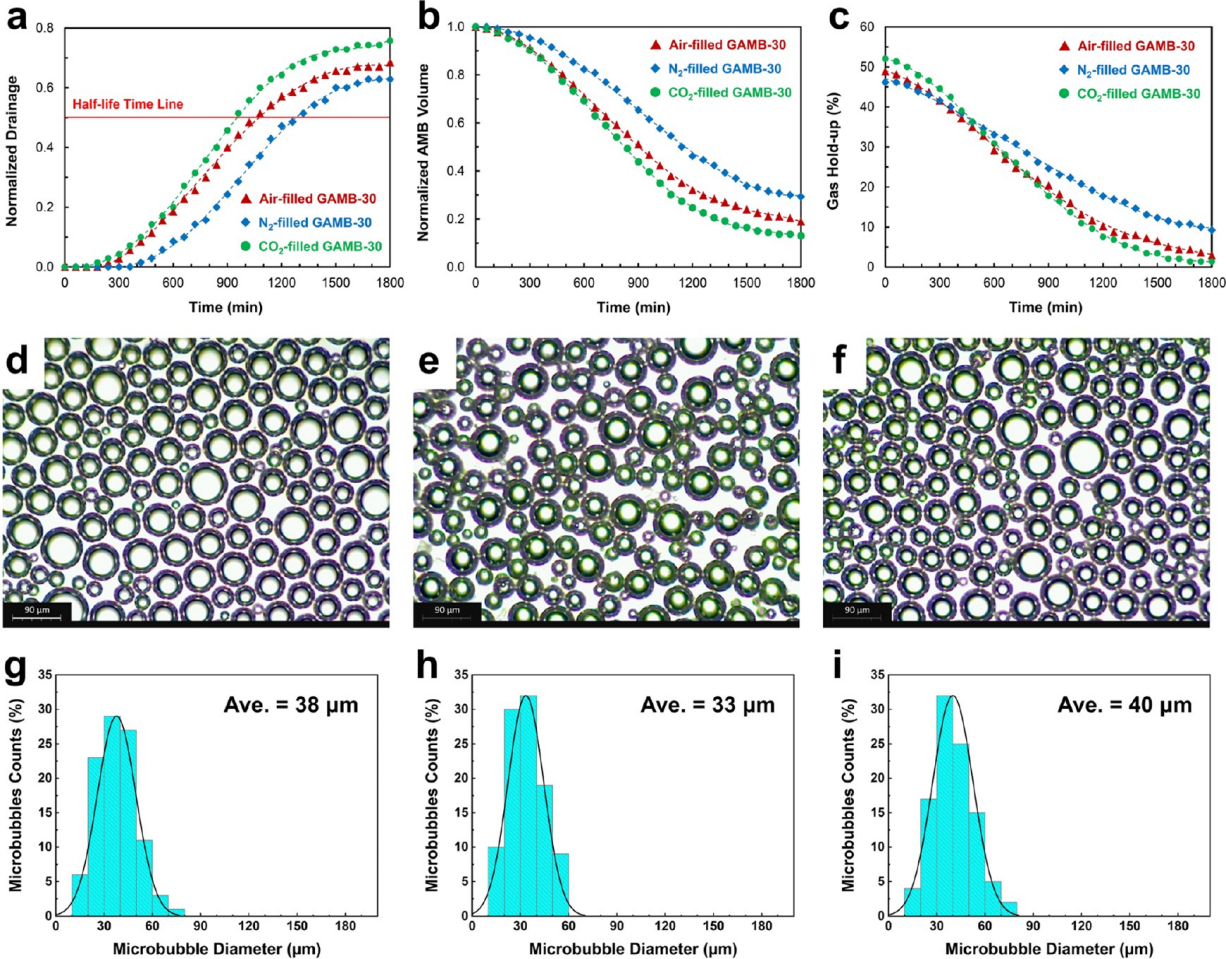
Effect of core gas type on physical properties of graphene-loaded aphron microbubbles. Time-behavior of (a) normalized drainage liquid, (b) normalized microbubble volume, and (c) gas hold-up. Optical micrograph and microbubble size distribution of air-filled (d, g), N_2_-filled (e, h), and CO_2_-filled (f, i) microbubbles. Experiments were carried out at 0.30 wt % PrGO under atmospheric pressure and constant temperature conditions of 26–28 °C.

Figure 5 illustrates the evolution of aphronic parameters over time and optical microscopic images alongside the corresponding size distribution of microbubbles filled with air, N_2_, and CO_2_ gases.

Notably, microbubble stability exhibits distinct behavior based on the core gas type. N_2_ gas yields the highest stability, while CO_2_ results in the lowest stability. The average sizes of microbubbles filled with air and N_2_ and CO_2_ gases were measured at 38, 33, and 40 μm, respectively. These variations in stability and size are attributed to differences in gas solubility and diffusion rates, which impact the growth and coalescence of microbubbles. The CO_2_ gas, with its higher solubility,^60^ leads to faster coalescence and growth, resulting in larger mean sizes compared to air and N_2_.

Microbubble shrinkage, a critical characteristic compared to regular bubbles, occurs due to gas diffusion from the trapped microbubble gas into the surrounding solution. The physicochemical properties of the gas significantly influence this process.^61^ Laplace pressure drives microbubble shrinkage, emphasizing the need to minimize gas mass transfer from the microbubble to the solution. The shell acts as a barrier, enhancing the stability by reducing gas permeability. The overall mass transfer coefficient, determined by gas properties (diffusion coefficient and Ostwald coefficient), directly affects the microbubble stability. Lower coefficients correlate with better shell impermeability.^62^ CO_2_, with its higher solubility and Ostwald coefficient, diffuses rapidly from microbubbles to the surrounding solution.^63^ Consequently, CO_2_ microbubbles exhibit a shorter half-life time. Initially, CO_2_ traps more gas than the other two gases, but dissolution eventually reduces this amount due to its greater solubility in water (Figure 5c).

Our results suggest that N_2_ and air are optimal choices for ultralight aphron drilling fluids, while CO_2_ is ideal for enhanced oil recovery and geological CO_2_ storage applications.

From an operational perspective, utilizing N_2_ gas may require additional equipment and considerations for the N_2_ gas supply and injection at the wellhead, which should be taken into account for economic feasibility.

### 3.4 Aphronic Water-Based Drilling Fluid Characterization

#### 3.4.1 Rheological Studies

The quantitative description of aphronic water-based fluid behavior relies on the concepts of shear stress and shear rate, as well as their measurement. Aphronic drilling fluids exhibit non-Newtonian behavior, varying with cutting rates and being strongly influenced by applied cutting rates. For an aphron-based fluid to maintain stability and function effectively as an aphronic fluid, it must possess the appropriate rheological properties. These properties include the ability to suspend microbubbles, prevent water molecule penetration in the microbubble membrane, and hinder molecular gas from entering the continuous phase. The stability of the aphron-based fluid structure is crucially dependent on its rheological properties.

**Figure 6:**
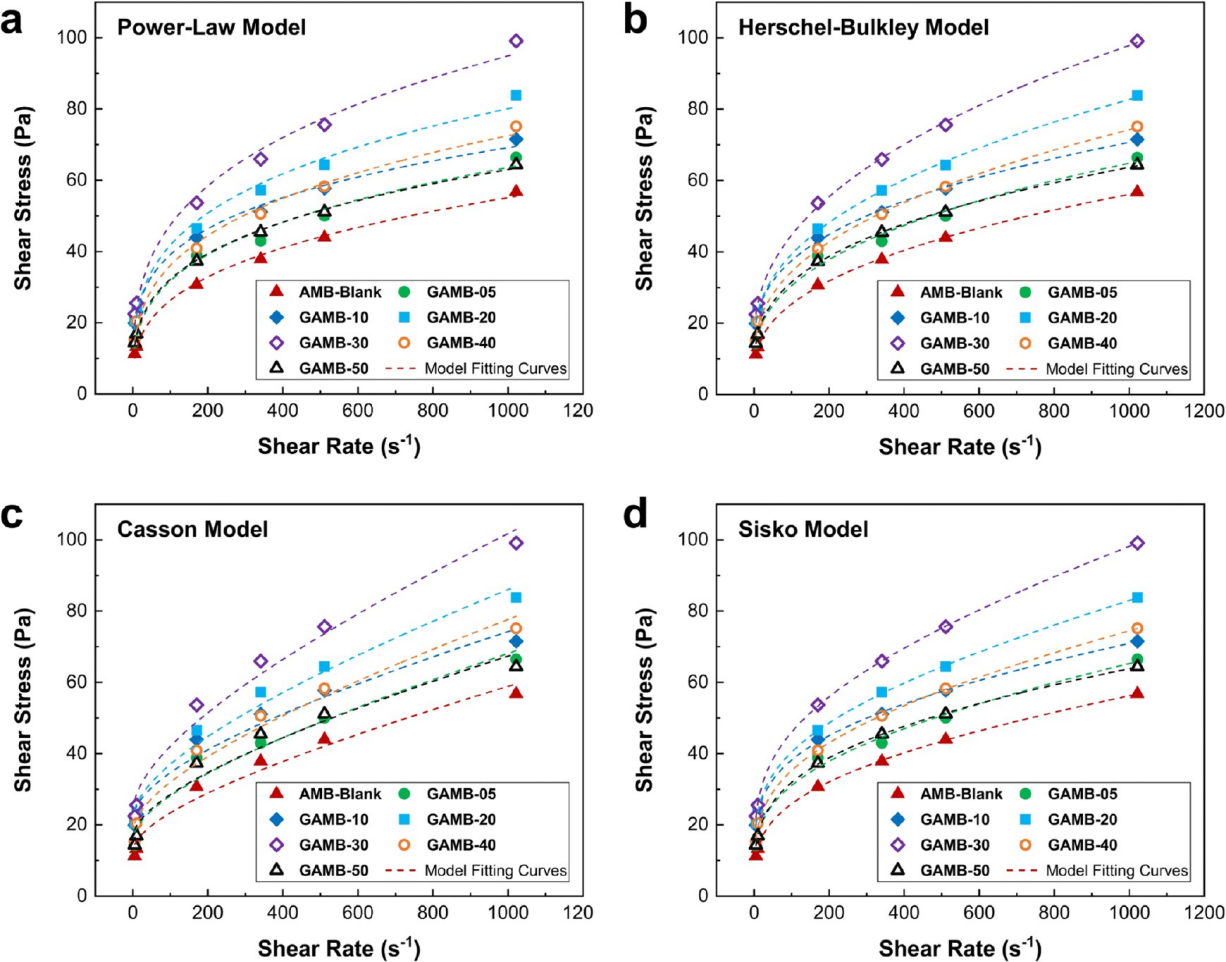
Rheological model fitting curves of the graphene-loaded AMB water-based drilling fluids for different concentrations of PrGO. (a) Power–Law model, (b) Herschel–Bulkley model, (c) Casson model, and (d) Sisko model.

The viscosity of the aphronic water-based drilling fluids was evaluated using a Fann 35 viscometer with a heater cup. Dial readings were taken at various rpm (600, 300, 200, 100, 6, and 3) and a constant temperature of 120 F in accordance with API standards. These readings were then converted to the shear stress and shear rate for analysis. To elucidate the behavior of aphron-based drilling fluids, shear stress (τ) and shear rate (γ) values were analyzed using four different rheological models, Power-Law, Herschel–Bulkley, Casson, and Sisko, according to eqs 3–6. The experimental data were then fitted to these rheological models using OriginPro software.
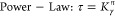
3
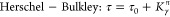
4
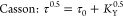
5
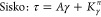
6where τ_0_ is yield point, *K* is the consistency index, and *n* is the flow behavior index. The application of these models to the relationship between shear rate and shear stress at various concentrations of PrGO is illustrated in Figure 6, with accompanying fitting parameters detailed in Table 1. A lower *R*^2^ value signifies reduced accuracy in describing the rheological behavior of aphronic water-based drilling fluids. The Herschel–Bulkley and Sisko models exhibited *R*^2^ values close to 1 across different concentrations of PrGO, indicating a superior fit to experimental data. Microscopic observations revealed that increasing the concentration of PrGO resulted in the formation of smaller bubbles, consequently elevating shear stress and viscosity in aphron-based fluids. Notably, sample GAMB-30, with a 0.3 wt % concentration of PrGO, exhibited improved rheological properties compared to other samples, as depicted in Figure 6.

**Table 1:** Rheological Fitting Parameters of the Graphene-Loaded AMB Water-Based Drilling Fluids for Different Concentrations of PrGO^a^

			PrGO concentration (wt %)					
model		blank	0.05	0.10	0.20	0.30	0.40	0.50
Power–Law	*K*	6.0 ± 0.5	7.9 ± 1.1	12.5 ± 0.9	11.4 ± 1.3	11.8 ± 1.5	8.9 ± 0.9	8.4 ± 0.4
	*n*	0.32 ± 0.01	0.30 ± 0.02	0.25 ± 0.01	0.28 ± 0.02	0.30 ± 0.02	0.30 ± 0.02	0.29 ± 0.01
	*R*^2^	0.9968	0.9886	0.9947	0.9900	0.9908	0.9936	0.9987
Herschel–Bulkley	τ_0_	5.2 ± 0.7	7.2 ± 4.7	10.7 ± 2.0	13.6 ± 1.3	14.6 ± 1.4	9.0 ± 2.2	4.5 ± 1.1
	*K*	3.2 ± 0.3	3.8 ± 2.3	5.4 ± 1.1	3.6 ± 0.6	3.9 ± 0.6	3.8 ± 1.0	5.6 ± 0.6
	*n*	0.40 ± 0.01	0.39 ± 0.08	0.35 ± 0.03	0.43 ± 0.02	0.44 ± 0.02	0.41 ± 0.03	0.34 ± 0.01
	*R*^2^	0.9997	0.9922	0.9991	0.9994	0.9995	0.9985	0.9998
Casson	τ_0_	3.5 ± 0.3	3.9 ± 0.3	4.6 ± 0.3	4.6 ± 0.2	4.8 ± 0.3	4.2 ± 0.3	4.0 ± 0.3
	*K*	0.13 ± 0.01	0.14 ± 0.01	0.13 ± 0.01	0.15 ± 0.01	0.17 ± 0.01	0.15 ± 0.01	0.13 ± 0.01
	*R*^2^	0.9717	0.9665	0.9669	0.9801	0.9810	0.9741	0.9604
Sisko	*A*	0.009 ± 0.001	0.013 ± 0.007	0.011 ± 0.002	0.020 ± 0.001	0.024 ± 0.001	0.015 ± 0.003	0.006 ± 0.001
	*K*	7.1 ± 0.2	9.6 ± 1.5	14.1 ± 0.4	14.2 ± 0.3	15.2 ± 0.3	10.8 ± 0.6	9.2 ± 0.2
	*n*	0.27 ± 0.01	0.25 ± 0.04	0.21 ± 0.01	0.22 ± 0.01	0.23 ± 0.01	0.25 ± 0.01	0.27 ± 0.01
	*R*^2^	0.9999	0.9940	0.9996	0.9999	0.9999	0.9992	0.9999

a The behavior of the prepared fluids is characterized by the parameters *K* (consistency coefficient) and *n* (flow behavior index). All samples exhibit a value of *n* less than 1, classifying them as non-Newtonian fluids, and with decreasing *n*, the shear-thinning ability of the fluid increases.

However, surpassing the optimal PrGO concentration did not yield favorable effects on the rheological properties of aphron-based drilling fluids. An elevated concentration of PrGO may result in oversaturation of the fluid with graphene nanosheets, thereby impeding the formation of small bubbles. The presence of smaller bubbles is advantageous for enhancing viscosity and flow characteristics, potentially diminishing the efficacy of microbubble formation on rheological properties.^64^ Furthermore, the heightened viscosity of aphronic water-based drilling fluids leads to delayed gravity drainage and thickening of the liquid film around the core, thereby enhancing the stability of microbubbles.

The findings obtained from the Sisko model analysis reveal that the parameter “*n*” in varying concentrations of PrGO is consistently lower than that of the control sample, with the lowest value observed at a concentration of 0.10 wt %. A decrease in the n index indicates a more non-Newtonian behavior of the fluid, resulting in a transition toward laminar flow and promoting shear-thinning characteristics. Shear thinning is a critical property of high-quality drilling fluids, as it enhances fluid performance during drilling operations. Furthermore, the parameter “*K*”, which represents effective viscosity, shows a substantial increase with the inclusion of PrGO nanosheets, peaking at a concentration of 0.30 wt %. This escalation in the *K* index can enhance fluid efficiency in hole cleaning and suspension of drilling fluid components.

#### 3.4.2 Filtration Loss

The mechanism of fluid invasion control of graphene-loaded AMBs is a complex process that plays an important role in maintaining well stability, preventing formation damage, and optimizing drilling operations in the oil and gas industry. When aphron-based drilling fluid enters a formation, microbubbles with graphene-reinforced shells and improved elasticity properties play a key role in creating a microenvironment that isolates the borehole from formation pressures. By improving the hydrophobic layer of the AMB shell by incorporation of PrGO nanosheets, the capillary pressure can play an effective role in controlling fluid invasion into the permeable and porous network of the low-pressure formation and improve the sealing effectiveness of AMB.

Enhanced aphrons actually have a precompressed structure when drilling in depleted or low-pressure formations due to the pressure of the fluid column and form a high-energy microenvironment. When the well pressure exceeds the formation pressure, the AMBs migrate from the well to the formation with a pressure gradient. The AMBs move to the front of the fluid due to the pressure difference, individually bridge the pores, and gather in the fractures and larger openings inside the formation and create an agglomeration of bubbles. With the release of the stored energy, the microbubble expansion process begins, and this process continues until the internal and external pressures are balanced. One of the key properties of drilling fluids that strongly affects their performance in this field is low shear rate viscosity (LSRV), which indicates the fluid’s resistance to flow under low shear conditions.

The LSRV measurements of the graphene-loaded AMB water-based drilling fluids are presented in Table S2, Supporting Information. The data demonstrate a significant increase in LSRV when graphene is incorporated into the AMB shell, such that at a concentration of 0.30 wt %, a staggering value of 224 000 cP was achieved at a speed of 0.3 rpm. This behavior can be attributed to the unique structure formed by the assembly of a multilayer shell comprising graphene sheets, surfactant molecules, and polymer chains. The presence of graphene sheets facilitates interactions between these components, leading to enhanced LSRV properties of the aphron-based drilling fluid. A high LSRV is critical in controlling the flow of drilling fluids into the formation during drilling operations, as it helps to maintain well integrity and prevent fluid loss.

**Figure 7:**
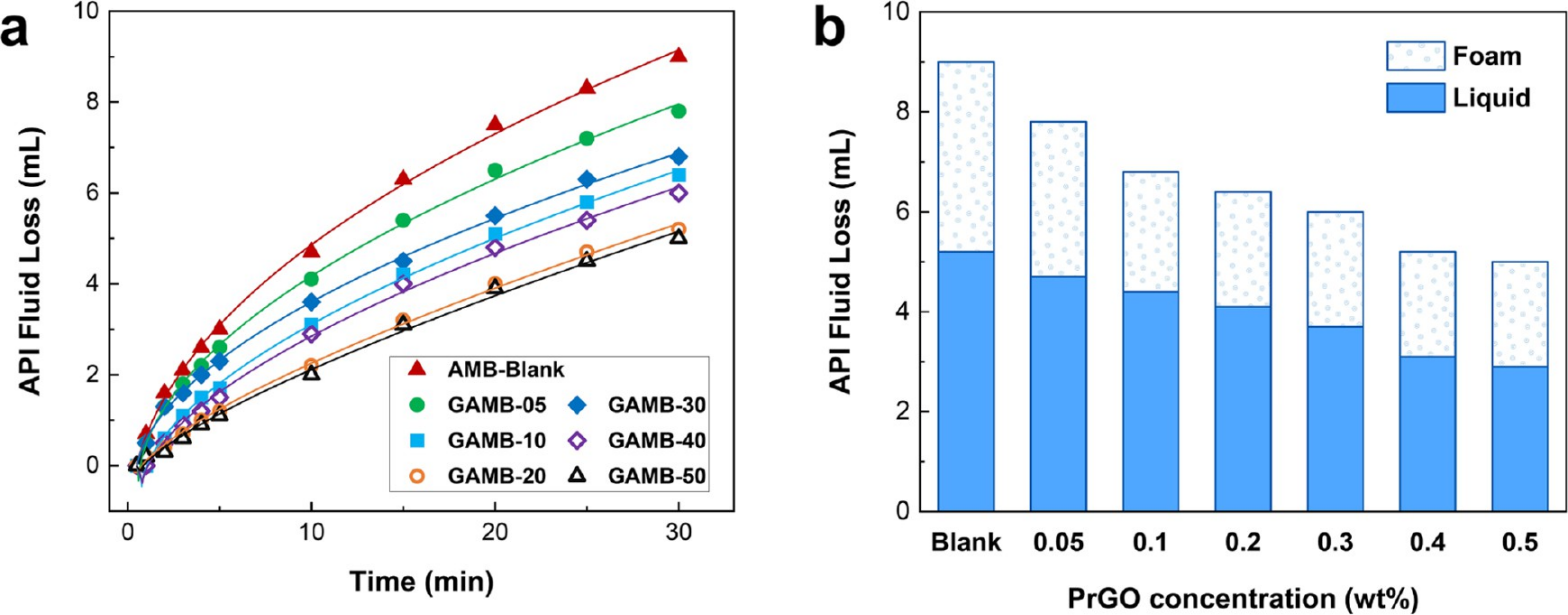
Filtration test results of the graphene-loaded AMB water-based drilling fluids for different concentrations of PrGO. (a) Filtration behavior with time, and (b) total fluid loss volume containing water and foam phases after 30 min. The filtration test was conducted using a multiunit filter press apparatus (LPLT, Fann) in accordance with the API filter press standard test procedure. The prepared drilling fluids were contained in a stainless steel vessel with a bottom outlet and subjected to a pressure of 100 psi (0.69 MPa). The amount of fluid loss was measured over a 30 min period, with data recorded every minute for the first 5 min and then at 5 min intervals thereafter.

Filtration loss is a critical parameter in drilling fluid design and performance evaluation, as it directly influences wellbore stability, formation damage, and overall drilling efficiency. The filtration test results of aphronic drilling fluids based on graphene-loaded microbubbles are given in Figure 7. Filtration behavior with time can be seen in Figure 7a, and the addition of PrGO nanosheets significantly reduces the filtration loss of the fluids. The filtration liquid of aphronic drilling fluid is composed of two parts, the lower part of freshwater and the upper part of the foam, and the fluid loss recorded is the total fluid loss, that is, the total volume of water and foam (Figure 7b). At a concentration of 0.05 wt %, the filtration loss is reduced by 13% compared to the base fluid without PrGO, and with further increasing the concentration, its effects on the fluid loss control are enhanced so that increasing the concentration to 0.50 wt % further decreases the filtration loss by 45%.

Also, to study filtration properties, the microstructure of fluid cakes was observed by optical microscope, as shown in Figure S2, Supporting Information. In the OM images of graphene-loaded microbubbles, the surface of the cake was smooth and dense, and there were some microbubbles. However, significant microbubbles with small size and narrow size distribution were observed in the cake of the sample with 0.3 wt % graphene.

The filtration control mechanism of graphene-loaded microbubbles is a complex process that involves the interaction of various factors. Microbubbles, with their low front pressure and flexible properties, can expand and aggregate before they enter the base phase of the fluid. This allows them to quickly fill the openings of filter pores and reorganize within the microenvironment, effectively preventing fluid loss. This process of microbubble plugging optimizes the filtration control of the aphronic drilling fluid. The decrease in the filtration losses observed can be attributed to two primary factors. First, the strong absorption of PrGO nanosheets within the microbubble shell leads to their stabilization in a smaller size. This results in enhanced resistance to filtration pressure, reduced liquid drainage, and, ultimately, less liquid loss during the filtration process. Second, the higher concentrations of PrGO play a significant role in reducing filtration losses. When the concentration exceeds a critical value and is absorbed within the microbubble shell, the remaining graphene sheets are dispersed in the continuous phase. The increase in dispersion of excessive PrGO nanosheets results in the formation of a more cohesive cake with reduced permeability and increased thickness up to approximately 2.5 times the blank sample (see Figure S2h, Supporting Information). Consequently, this leads to a decrease in the overall filtration rate.

**Figure 8:**
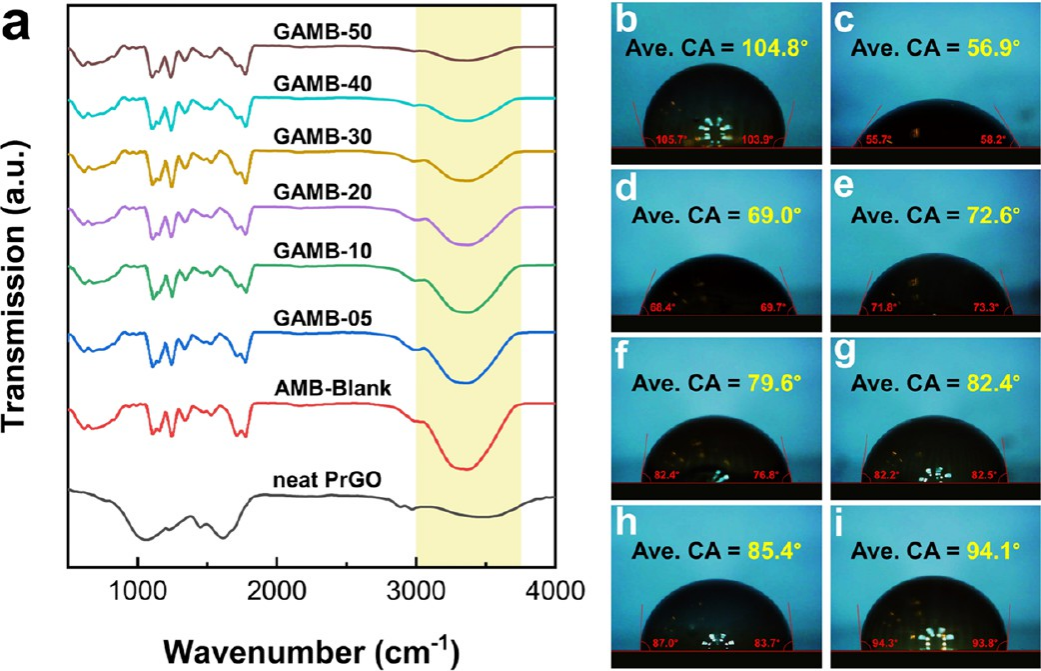
Physicochemical characterization of aphronic fluid filtration cakes. (a) FTIR spectra and contact angle of (b) neat PrGO, (c) blank, (d) 0.05 wt %, (e) 0.10 wt %, (f) 0.20 wt %, (g) 0.30 wt %, (h) 0.40 wt %, and (i) 0.50 wt % PrGO.

To thoroughly investigate the impact of PrGO concentration on the bonding and physicochemical properties of filter cakes, FTIR spectroscopy and contact angle measurements were conducted on cakes prepared with varying concentrations of PrGO nanosheets, with the findings presented in Figure 8. The FTIR spectra (Figure 8a) revealed multiple peaks below 2000 cm^–1^, indicative of the characteristic features of polysaccharides predominantly found in the cake samples containing starch and xanthan gum.^65^

In the absence of PrGO (Blank sample), the FTIR analysis identified prominent peaks at 1110 and 1158 cm^–1^, corresponding to the C–O bonds in the anhydrous glucose ring. Additionally, a significant peak at 1247 cm^–1^ and a weaker peak at 2985 cm^–1^ were attributed to C–H wagging and stretching, respectively. The peak at 1532 cm^–1^ was linked to the symmetric and asymmetric stretching vibrations of C=O groups in the acetyl group of the gum.^66^ A broad absorption peak observed between 3047 and 3742 cm^–1^ indicated the presence of hydrogen-bonded −OH groups.^67^

The graphene-loaded aphron drilling fluid cakes exhibited all of the major characteristics of the Blank samples, with a notable difference observed in the decrease in intensity of the band corresponding to the OH group (at 3358 cm^–1^) with higher PrGO incorporation. This variation underscores the increasing presence of graphene in the filter cake as its concentration rises, highlighting an elevation in the surface hydrophobicity with the incorporation of higher quantities of graphene nanosheets into the cake matrix. These results were confirmed by measuring the contact angle on the surface of the obtained cakes (Figure 8b–i). According to the results, with the increase in graphene concentration, the contact angle increased from 56° for the raw sample to 94° for the sample with 0.5 wt % of PrGO, which indicates an increase in the hydrophobicity of the cake surface.

Table 2 presents a comparison of the findings of the current research with those discussed in the existing literature. The key highlight of the study is the utilization of a structure tailored specifically for the application environment. In fact, By optimizing the absorption and self-assembly of amphiphilic PrGO nanosheets within the microbubble shell, the research has achieved a more uniform size distribution with a reduced average size and increased stability in the aphronic microbubbles produced. The findings were achieved using a notably reduced concentration of enhancer materials in comparison to what is typically reported in the current body of research. The innovative structure developed in this study has notably enhanced the rheological properties, filtration efficiency, and viscosity at lower shear rates of aphronic water-based drilling fluid, surpassing the results reported in previous studies.

**Table 2:** Comparison between Previous Literature Data and the Results of This Study

ref	year	fluid category	improvement material	concentration	average microbubble size (μm)	microbubble size distribution	AV (cP)	FL (mL)	τ_1/2_ (min)	LSRV (cP)
(25)	2023	water-based	nano SiO_2_	3%			101		720	
(68)	2023	water-based	sugar cane molasses	12%	51.9	10–100<	52	19.5	10.6	
(26)	2021	water-based	modified starch	1%	93.3	88.4% in 10–150 μm	102	13.2	<90	16 396
(27)	2021	water-based	alkyl glycine	3%	87.7	91.5% in 10–150 μm	112.5	6.5	98.3	23 4000
(18)	2021	water-based	grafted xanthan copolymer	1.5%	81.7	97.2% in 10–150 μm	150 <	10<		30 394
(28)	2021	water-based	grafted starch copolymer	2%	139.1	40–300 μm		7<	7200<	204 000
(36)	2020	water-based	silica and fumed-silica nanoparticles	0.429%	70	20–100	50–70	7<	<200	
(17)	2020	water-based	attapulgite	3%	84.8	48.48% in 50–100 μm	41	9		102 000
(69)	2017	kerosene-based	hydrophobic nano SiO_2_	1%				80	<350	12 745
			hydrophobic nano clay	0.8%				71	<350	14 578
(70)	2017	water-based	glycyrrhiza glabra	1.14%	65	10–200 μm	67.5	∼16		
this study	2024	water-based	partially reduced graphene oxide (PrGO) nanosheets	0.3%	37.8	100% in 5–80 μm	97	6 (3.7 mL of liquid and 2.3 mL of foam)	1050	224 000

Furthermore, the study also highlights the potential use of graphene-loaded aphron microbubbles in carbon geological storage. Carbon geological storage is a technology used to capture and store carbon dioxide emissions from industrial processes to mitigate climate change. By leveraging the unique properties of graphene-loaded aphron microbubbles, the efficiency and effectiveness of leak-free carbon geological storage processes can be enhanced. The microbubbles can help in trapping and transporting carbon dioxide in underground geological formations, preventing its release into the atmosphere. In the future, the findings of this research study could pave the way for the development and commercialization of advanced drilling fluid formulations and carbon geological storage technologies that utilize microbubbles-based fluids. These innovative solutions have the potential to significantly impact the oil and gas industry by improving drilling efficiency and reducing environmental impact through enhanced carbon capture and storage capabilities. Further research and development in this area could lead to practical applications that benefit both industry and the environment.

## 4 Conclusions

In conclusion, we have shown that the incorporation of PrGO nanosheets into the shell of aphron microbubbles presents a novel and innovative approach to enhancing the stability, size distribution, and overall performance of aphronic water-based drilling fluids. PrGO nanosheets, with their unique amphiphilic properties, play a crucial role in improving the mechanical strength of the microbubble shell, preventing coalescence and Ostwald ripening and increasing the resistance to gas diffusion, thereby enhancing the stability of aphron microbubbles. Through this research, it has been observed that PrGO nanosheets significantly impact the stability, size distribution, rheological properties, filtration loss, and hydrophobicity of the drilling fluid cakes, contributing to the improved performance and efficiency of the water-based drilling fluid.

The high surface area of PrGO nanosheets allows for a better interaction between surfactant molecules and PrGO, resulting in a more stable microbubble structure. The incorporation of PrGO nanosheets in the AMB shell has resulted in a more homogeneous size distribution and a decrease in microbubble size of up to 60% at a concentration of 0.3 wt %. Additionally, by enhancement of resistance to drainage and gas diffusion within the microbubble structure, the half-life time (stability) has markedly increased by approximately 120%. The concentration of PrGO plays a critical role in determining the effectiveness of the microbubble shell, with an optimal concentration of 0.30 wt % yielding the best results in terms of stability and size distribution.

The addition of PrGO nanosheets has also influenced the rheological properties of aphronic water-based drilling fluids, affecting shear stress, shear rate, and viscosity. Different rheological models show varied degrees of accuracy in describing the behavior of aphron-based fluids, with the Herschel–Bulkley and Sisko models demonstrating a superior fit. The increased viscosity of the drilling fluid due to PrGO leads to enhanced stability, delayed drainage, and improved resistance to collapse.

Furthermore, the inclusion of PrGO nanosheets has improved filtration control in aphronic water-based drilling fluids, reducing filtration loss and enhancing stability. The structural properties of PrGO nanosheets act as an effective barrier and bridging agent to prevent the migration of solids and particles across the filter medium.

The results of this study suggest that the use of graphene-loaded aphron microbubbles filled by air or N_2_ gas could lead to the development of lightweight and ultralightweight water-based drilling fluids that offer substantial benefits to the oil and gas industry. Additionally, the graphene-loaded aphron microbubbles filled with CO_2_ gas show promise in carbon capture and storage operations, providing an efficient agent for trapping and transporting CO_2_ within the microbubble core. This functionality is crucial for underground CO_2_ storage operations, contributing to the reduction of carbon emissions and addressing climate change challenges.

## Supporting Information Available

Self-assembly mechanism of graphene-loaded aphron microbubbles formation; optical micrographs of aphronic fluid filtration cake; concentrations of the used materials in the formulation of the AMB water-based drilling fluid; and low shear rate viscosity (LSRV) of the graphene-loaded AMB water-based drilling fluids at different concentrations of PrGO (PDF)

## References

[ref1] Orun C. B.; Akpabio J. U.; Agwu O. E. Drilling fluid design for depleted zone drilling: An integrated review of laboratory, field, modelling and cost studies. Geoenergy Sci. Eng. 2023, 226, 211706 10.1016/j.geoen.2023.211706.

[ref2] Collins I. R.; Floriano D. C.; Paevskiy I.; Wee J.; Boek E. S. Transition from oil & gas drilling fluids to geothermal drilling fluids. Geoenergy Sci. Eng. 2023, 212543 10.1016/j.geoen.2023.212543.

[ref3] Molaei A.; Waters K. Aphron applications–a review of recent and current research. Adv. Colloid Interface Sci. 2015, 216, 36–54. 10.1016/j.cis.2014.12.001. 25578407

[ref4] Shivhare S.; Kuru E. A study of the pore-blocking ability and formation damage characteristics of oil-based colloidal gas aphron drilling fluids. J. Pet. Sci. Eng. 2014, 122, 257–265. 10.1016/j.petrol.2014.07.018.

[ref5] Geng X.; Hu X.; Jia X. Recirculated aphron-based drilling fluids. J. Pet. Explor. Prod. Technol. 2014, 4, 337–342. 10.1007/s13202-013-0094-6.

[ref6] Deng Q.; Mi J.; Dong J.; Chen Y.; Chen L.; He J.; Zhou J. Superiorly stable three-layer air microbubbles generated by versatile ethanol–water exchange for contrast-enhanced ultrasound theranostics. ACS Nano 2023, 17 (1), 263–274. 10.1021/acsnano.2c07300. 36354372

[ref7] Pal P.; W Hasan S.; Abu Haija M.; Sillanpää M.; Banat F. Colloidal gas aphrons for biotechnology applications: a mini review. Crit. Rev. Biotechnol. 2023, 43 (7), 971–981. 10.1080/07388551.2022.2092716. 35968911

[ref8] Park J. I.; Jagadeesan D.; Williams R.; Oakden W.; Chung S.; Stanisz G. J.; Kumacheva E. Microbubbles loaded with nanoparticles: a route to multiple imaging modalities. ACS Nano 2010, 4 (11), 6579–6586. 10.1021/nn102248g. 20968309

[ref9] Chen M.; Liang X.; Gao C.; Zhao R.; Zhang N.; Wang S.; Chen W.; Zhao B.; Wang J.; Dai Z. Ultrasound triggered conversion of porphyrin/camptothecin-fluoroxyuridine triad microbubbles into nanoparticles overcomes multidrug resistance in colorectal cancer. ACS Nano 2018, 12 (7), 7312–7326. 10.1021/acsnano.8b03674. 29901986

[ref10] Ullah M.; Kodam S. P.; Mu Q.; Akbar A. Microbubbles versus extracellular vesicles as therapeutic cargo for targeting drug delivery. ACS Nano 2021, 15 (3), 3612–3620. 10.1021/acsnano.0c10689. 33666429

[ref11] Ward K.; Taylor A.; Mohammed A.; Stuckey D. C. Current applications of Colloidal Liquid Aphrons: Predispersed solvent extraction, enzyme immobilization and drug delivery. Adv. Colloid Interface Sci. 2020, 275, 102079 10.1016/j.cis.2019.102079. 31787216

[ref12] Akhlaghi M. H.; Naderi M. Stabilized Colloidal Aphron/Graphene Derivatives Hybrid Fluids. US11,891,493B2, 2024.

[ref13] Dermiki M.; Garrard I. J.; Jauregi P. Selective separation of dyes by colloidal gas aphrons: Conventional flotation vs countercurrent chromatography. Sep. Purif. Technol. 2021, 279, 119770 10.1016/j.seppur.2021.119770.

[ref14] Priyanka M.; Saravanakumar M. A sustainable approach for removal of microplastics from water matrix using Colloidal Gas Aphrons: New insights on flotation potential and interfacial mechanism. J. Cleaner Prod. 2022, 334, 130198 10.1016/j.jclepro.2021.130198.

[ref15] Keshavarzi B.; Javadi A.; Bahramian A.; Miller R. Formation and stability of colloidal gas aphron based drilling fluid considering dynamic surface properties. J. Pet. Sci. Eng. 2019, 174, 468–475. 10.1016/j.petrol.2018.11.057.

[ref16] Spinelli L. S.; Neto G. R.; Freire L. F.; Monteiro V.; Lomba R.; Michel R.; Lucas E. Synthetic-based aphrons: correlation between properties and filtrate reduction performance. Colloids Surf., A 2010, 353 (1), 57–63. 10.1016/j.colsurfa.2009.10.017.

[ref17] Zhu W.; Zheng X.; Li G. Micro-bubbles size, rheological and filtration characteristics of Colloidal Gas Aphron (CGA) drilling fluids for high temperature well: Role of attapulgite. J. Pet. Sci. Eng. 2020, 186, 106683 10.1016/j.petrol.2019.106683.

[ref18] Zhu W.; Zheng X.; Shi J.; Wang Y. A high-temperature resistant colloid gas aphron drilling fluid system prepared by using a novel graft copolymer xanthan gum-AA/AM/AMPS. J. Pet. Sci. Eng. 2021, 205, 108821 10.1016/j.petrol.2021.108821.

[ref19] Li X.; Peng B.; Liu Q.; Liu J.; Shang L. Micro and nanobubbles technologies as a new horizon for CO_2_-EOR and CO_2_ geological storage techniques: A review. Fuel 2023, 341, 127661 10.1016/j.fuel.2023.127661.

[ref20] Buapuean T.; Jarudilokkul S. Synthesis of mesoporous TiO_2_ with colloidal gas aphrons, colloidal liquid aphrons, and colloidal emulsion aphrons for dye-sensitized solar cells. Mater. Today Chem. 2020, 16, 100235 10.1016/j.mtchem.2019.100235.

[ref21] Wang N. N.; Wang Y. P.; Zhang D.; Tang H. M. Micro foam drilling fluid system performance research and application. Adv. Mater. Res. 2013, 868, 601–605. 10.4028/www.scientific.net/AMR.868.601.

[ref22] Sun Q.; Xu B. Application of micro-foam drilling fluid technology in Haita area. Nat. Sci. 2012, 04, 438 10.4236/ns.2012.47059.

[ref23] Sakhaei Z.; Ghorbani-Saadatabadi N.; Escrochi M.; Riazi M. Mechanistic insight into the colloidal gas aphrons stability in the presence of petroleum hydrocarbons. Fuel 2024, 368, 131576 10.1016/j.fuel.2024.131576.

[ref24] Bjorndalen N.; Kuru E. Stability of Microbubble Based Drilling Fluids Under Downhole Conditions. In PETSOC Canadian International Petroleum Conference; PETSOC, 2006; pp 2006–2079.

[ref25] Zhu W.; Wang B.; Zheng X. Preparation and Foam Stabilization Mechanism of an Ultrahigh-Temperature Colloidal Gas Aphron (CGA) System Based on Nano-SiO_2_. ACS Omega 2023, 8 (48), 46091–46100. 10.1021/acsomega.3c07131. 38075745 PMC10702178

[ref26] Zhu W.; Zheng X. Application of modified starch in high-temperature-resistant colloidal gas aphron (CGA) drilling fluids. J. Polym. Eng. 2021, 41 (6), 458–466. 10.1515/polyeng-2021-0042.

[ref27] Zhu W.; Zheng X. Alkyl Glycine Surfactant: An Efficient High-Temperature Resistant and Biodegradable Foaming Agent for Colloidal Gas Aphron (CGA) Drilling Fluid. Pet. Chem. 2021, 61, 1305–1318. 10.1134/S0965544121110220.

[ref28] Zhu W.; Zheng X.; Shi J.; Wang Y. Grafted Starch Foam Stabilizer ESt-g-NAA for High-Temperature Resistant CGA Drilling Fluid via Inverse Emulsion Polymerization. Starch-Stärke 2021, 73 (9–10), 2000240 10.1002/star.202000240.

[ref29] Jauregi P.; Mitchell G. R.; Varley J. Colloidal gas aphrons (CGA): dispersion and structural features. AIChE J. 2000, 46 (1), 24–36. 10.1002/aic.690460105.

[ref30] Hashim M. A.; Mukhopadhyay S.; Gupta B. S.; Sahu J. N. Application of colloidal gas aphrons for pollution remediation. J. Chem. Technol. Biotechnol. 2012, 87 (3), 305–324. 10.1002/jctb.3691.

[ref31] Borden M. A.; Longo M. L. Dissolution behavior of lipid monolayer-coated, air-filled microbubbles: Effect of lipid hydrophobic chain length. Langmuir 2002, 18 (24), 9225–9233. 10.1021/la026082h.

[ref32] Borrelli M. J.; O’Brien Jr W. D.; Bernock L. J.; Williams H. R.; Hamilton E.; Wu J.; Oelze M. L.; Culp W. C. Production of uniformly sized serum albumin and dextrose microbubbles. Ultrason. Sonochem. 2012, 19 (1), 198–208. 10.1016/j.ultsonch.2011.05.010. 21689961 PMC3152625

[ref33] Eisenbrey J. R.; Wheatley M. A.; O’kane P.; Albala L.; Forsberg F. Surfactant Microbubbles and Process for Preparing and Methods of Using the Same. US11,305,013.2022.

[ref34] Bjerknes K.; Sontum P.; Smistad G.; Agerkvist I. Preparation of polymeric microbubbles: formulation studies and product characterisation. Int. J. Pharm. 1997, 158 (2), 129–136. 10.1016/S0378-5173(97)00228-7.

[ref35] Stocco A.; Drenckhan W.; Rio E.; Langevin D.; Binks B. P. Particle-stabilised foams: an interfacial study. Soft Matter 2009, 5 (11), 2215–2222. 10.1039/b901180c.

[ref36] Tabzar A.; Ziaee H.; Arabloo M.; Ghazanfari M. H. Physicochemical properties of nano-enhanced colloidal gas aphron (NCGA)-based fluids. Eur. Phys. J. Plus 2020, 135, 312 10.1140/epjp/s13360-020-00174-5.

[ref37] Arriaga L. R.; Drenckhan W.; Salonen A.; Rodrigues J. A.; Iniguez-Palomares R.; Rio E.; Langevin D. On the long-term stability of foams stabilised by mixtures of nano-particles and oppositely charged short chain surfactants. Soft Matter 2012, 8 (43), 11085–11097. 10.1039/c2sm26461g.

[ref38] Rio E.; Drenckhan W.; Salonen A.; Langevin D. Unusually stable liquid foams. Adv. Colloid Interface Sci. 2014, 205, 74–86. 10.1016/j.cis.2013.10.023. 24342735

[ref39] Wang B.; Li Z.; Wang C.; Signetti S.; Cunning B. V.; Wu X.; Huang Y.; Jiang Y.; Shi H.; Ryu S.; et al. Folding Large Graphene-on-Polymer Films Yields Laminated Composites with Enhanced Mechanical Performance. Adv. Mater. 2018, 30 (35), 1707449 10.1002/adma.201707449. 29992669

[ref40] Wang M.; Duan X.; Xu Y.; Duan X. Functional three-dimensional graphene/polymer composites. ACS Nano 2016, 10 (8), 7231–7247. 10.1021/acsnano.6b03349. 27403991

[ref41] Liu P.; Li X.; Min P.; Chang X.; Shu C.; Ding Y.; Yu Z.-Z. 3D lamellar-structured graphene aerogels for thermal interface composites with high through-plane thermal conductivity and fracture toughness. Nano-Micro Lett. 2021, 13, 22 10.1007/s40820-020-00548-5. PMC8187529 34138210

[ref42] Kusrini E.; Oktavianto F.; Usman A.; Mawarni D. P.; Alhamid M. I. Synthesis, characterization, and performance of graphene oxide and phosphorylated graphene oxide as additive in water-based drilling fluids. Appl. Surf. Sci. 2020, 506, 145005 10.1016/j.apsusc.2019.145005.

[ref43] Kusrini E.; Suhrowati A.; Usman A.; Khalil M.; Degirmenci V. Synthesis and characterization of graphite oxide, graphene oxide and reduced graphene oxide from graphite waste using modified Hummers’s method and zinc as reducing agent. Synthesis 2019, 10 (6), 1093–1104. 10.14716/ijtech.v10i6.3639.

[ref44] McCoy T. M.; Turpin G.; Teo B. M.; Tabor R. F. Graphene oxide: a surfactant or particle?. Curr. Opin. Colloid Interface Sci. 2019, 39, 98–109. 10.1016/j.cocis.2019.01.010.

[ref45] Gamot T. D.; Bhattacharyya A. R.; Sridhar T.; Beach F.; Tabor R. F.; Majumder M. Synthesis and stability of water-in-oil emulsion using partially reduced graphene oxide as a tailored surfactant. Langmuir 2017, 33 (39), 10311–10321. 10.1021/acs.langmuir.7b02320. 28872873

[ref46] Li J.; Feng Q.; Cui J.; Yuan Q.; Qiu H.; Gao S.; Yang J. Self-assembled graphene oxide microcapsules in Pickering emulsions for self-healing waterborne polyurethane coatings. Compos. Sci. Technol. 2017, 151, 282–290. 10.1016/j.compscitech.2017.07.031.

[ref47] Soltani T.; Lee B.-K. Low intensity-ultrasonic irradiation for highly efficient, eco-friendly and fast synthesis of graphene oxide. Ultrason. Sonochem. 2017, 38, 693–703. 10.1016/j.ultsonch.2016.08.010. 27622703

[ref48] Shadkam R.; Naderi M.; Ghazitabar A.; Asghari-Alamdari A.; Shateri S. Enhanced electrochemical performance of graphene aerogels by using combined reducing agents based on mild chemical reduction method. Ceram. Int. 2020, 46 (14), 22197–22207. 10.1016/j.ceramint.2020.05.297.

[ref49] Wasalathilake K. C.; Galpaya D. G.; Ayoko G. A.; Yan C. Understanding the structure-property relationships in hydrothermally reduced graphene oxide hydrogels. Carbon 2018, 137, 282–290. 10.1016/j.carbon.2018.05.036.

[ref50] Dimiev A. M.; Eigler S. Graphene Oxide: Fundamentals and Applications; John Wiley & Sons, 2016.

[ref51] Barrabino A.; Holt T.; Lindeberg E. An evaluation of graphene oxides as possible foam stabilizing agents for CO_2_ based enhanced oil recovery. Nanomaterials 2018, 8 (8), 603 10.3390/nano8080603. 30096822 PMC6116202

[ref52] Li H.; Xue S.; Shang Y.; Li J.; Zeng X. Research and application progress based on the interfacial properties of graphene oxide. Adv. Mater. Interfaces 2020, 7 (21), 2000881 10.1002/admi.202000881.

[ref53] Kim H.; Jang Y. R.; Yoo J.; Seo Y.-S.; Kim K.-Y.; Lee J.-S.; Park S.-D.; Kim C.-J.; Koo J. Morphology control of surfactant-assisted graphene oxide films at the liquid–gas interface. Langmuir 2014, 30 (8), 2170–2177. 10.1021/la403255q. 24499257

[ref54] McCoy T. M.; De Campo L.; Sokolova A. V.; Grillo I.; Izgorodina E. I.; Tabor R. F. Bulk properties of aqueous graphene oxide and reduced graphene oxide with surfactants and polymers: adsorption and stability. Phys. Chem. Chem. Phys. 2018, 20 (24), 16801–16816. 10.1039/C8CP02738B. 29888351

[ref55] Gravelle S.; Botto L. Adsorption of single and multiple graphene-oxide nanoparticles at a water–vapor interface. Langmuir 2021, 37 (45), 13322–13330. 10.1021/acs.langmuir.1c01902. 34723541

[ref56] Soleimani A.; Risselada H. J. Pure Graphene Acts as an “Entropic Surfactant” at the Octanol–Water Interface. ACS Nano 2023, 17 (14), 13554–13562. 10.1021/acsnano.3c02107. 37432037 PMC10373651

[ref57] Inoue S.; Kimura Y.; Uematsu Y. Ostwald ripening of aqueous microbubble solutions. J. Chem. Phys. 2022, 157 (24), 244704 10.1063/5.0128696. 36586988

[ref58] Yu Y.; Wang C.; Liu J.; Mao S.; Mehmani Y.; Xu K. Bubble coarsening kinetics in porous media. Geophys. Res. Lett. 2023, 50 (1), e2022GL100757 10.1029/2022GL100757.

[ref59] Stevenson P. Inter-bubble gas diffusion in liquid foam. Curr. Opin. Colloid Interface Sci. 2010, 15 (5), 374–381. 10.1016/j.cocis.2010.05.010.

[ref60] Nguyen Hai Le N.; Sugai Y.; Sasaki K. Investigation of stability of CO_2_ microbubbles—colloidal gas aphrons for enhanced oil recovery using definitive screening design. Colloids Interfaces 2020, 4 (2), 26 10.3390/colloids4020026.

[ref61] Lee M.; Lee E. Y.; Lee D.; Park B. J. Stabilization and fabrication of microbubbles: applications for medical purposes and functional materials. Soft Matter 2015, 11 (11), 2067–2079. 10.1039/C5SM00113G. 25698443

[ref62] Sridhar S.; Patel A.; Dalvi S. V. Estimation of storage stability of aqueous microbubble suspensions. Colloids Surf., A 2016, 489, 182–190. 10.1016/j.colsurfa.2015.10.044.

[ref63] Cadogan S. P.; Maitland G. C.; Trusler J. M. Diffusion coefficients of CO_2_ and N_2_ in water at temperatures between 298.15 and 423.15 K at pressures up to 45 MPa. J. Chem. Eng. Data 2014, 59 (2), 519–525. 10.1021/je401008s.

[ref64] Shams M. M.; Dong M.; Mahinpey N. Viscosity and rheological behavior of microbubbles in capillary tubes. AIChE J. 2014, 60 (7), 2660–2669. 10.1002/aic.14434.

[ref65] Shalviri A.; Liu Q.; Abdekhodaie M. J.; Wu X. Y. Novel modified starch–xanthan gum hydrogels for controlled drug delivery: Synthesis and characterization. Carbohydr. Polym. 2010, 79 (4), 898–907. 10.1016/j.carbpol.2009.10.016.

[ref66] Weber F. H.; Clerici M. T. P.; Collares-Queiroz F. P.; Chang Y. K. Interaction of guar and xanthan gums with starch in the gels obtained from normal, waxy and high-amylose corn starches. Starch-Stärke 2009, 61 (1), 28–34. 10.1002/star.200700655.

[ref67] Li R.; Liu C.; Ma J. Studies on the properties of graphene oxide-reinforced starch biocomposites. Carbohydr. Polym. 2011, 84 (1), 631–637. 10.1016/j.carbpol.2010.12.041.

[ref68] Saeedi Dehaghani A. H.; Elyaderani S. M. G. Experimental investigation of the impact of sugarcane molasses on the properties of colloidal gas aphron (CGA) drilling fluid. Petroleum 2023, 9 (2), 199–204. 10.1016/j.petlm.2021.07.004.

[ref69] Hassani A. H.; Ghazanfari M. H. Improvement of non-aqueous colloidal gas aphron-based drilling fluids properties: Role of hydrophobic nanoparticles. J. Nat. Gas Sci. Eng. 2017, 42, 1–12. 10.1016/j.jngse.2017.03.005.

[ref70] Ali Ahmadi M.; Galedarzadeh M.; Reza Shadizadeh S. Spotlight on the use of new natural surfactants in colloidal gas aphron (CGA) fluids: A mechanistic study. Eur. Phys. J. Plus 2017, 132, 519 10.1140/epjp/i2017-11792-1.

