# Supplementary material for: Graphene-Loaded Aphron Microbubbles for Enhanced Drilling Fluid Performance and Carbon Capture and Storage
Source: ACS Appl Nano Mater. 2024 Nov 11;7(22):26187–201. doi: 10.1021/acsanm.4c05693 (PMC11590059; doi:10.1021/acsanm.4c05693)
Supplement: Supplementary file 1 — an4c05693_si_001.pdf [file an4c05693_si_001.pdf]

Supporting Information

Graphene-Loaded Aphron Microbubbles for Enhanced Drilling Fluid Performance and Carbon Capture and Storage

Mohammad Hossein Akhlaghi[1,2], Malek Naderi[1,2,*], Mojtaba Abdi-Jalebi[3,*]

[1] Department of Materials and Metallurgical Engineering, Amirkabir University of Technology (Tehran Polytechnic), 1591634311, Tehran, Iran

[2] Graphene and Advanced Materials Laboratory (GAMLab), Innovation tower of Amirkabir University of Technology (Tehran Polytechnic), 1591634311, Tehran, Iran

[3] Institute for Materials Discovery, University College London, Malet Place, London, WC1E 7JE, United Kingdom

* To whom correspondence should be addressed. E-mail: m.jalebi@ucl.ac.uk (M.A.-J.), mnaderi@aut.ac.ir (M.N.);

This PDF file includes:

Supplementary Figures S1-S2

Supplementary Tables S1-S2

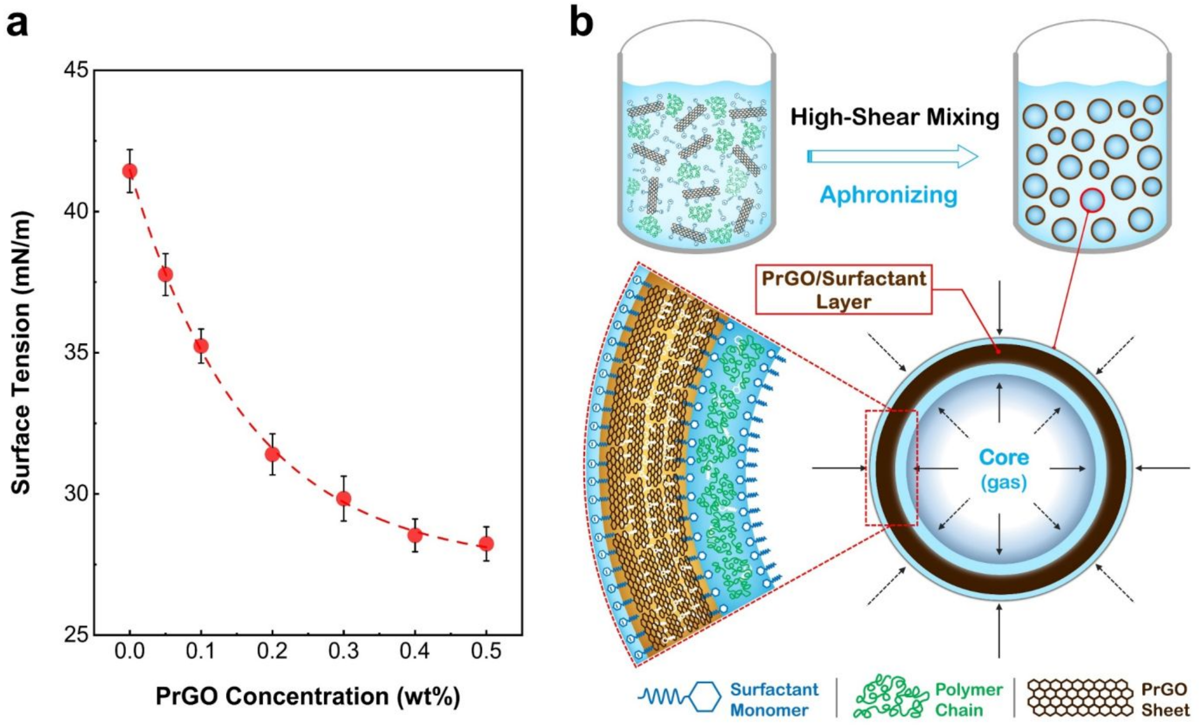

Figure S1. Self-assembly mechanism of graphene-loaded aphron microbubbles formation. a) Schematic of the self-assembly of shell layers, and **b**) The effect of PrGO concentration on surface tension.

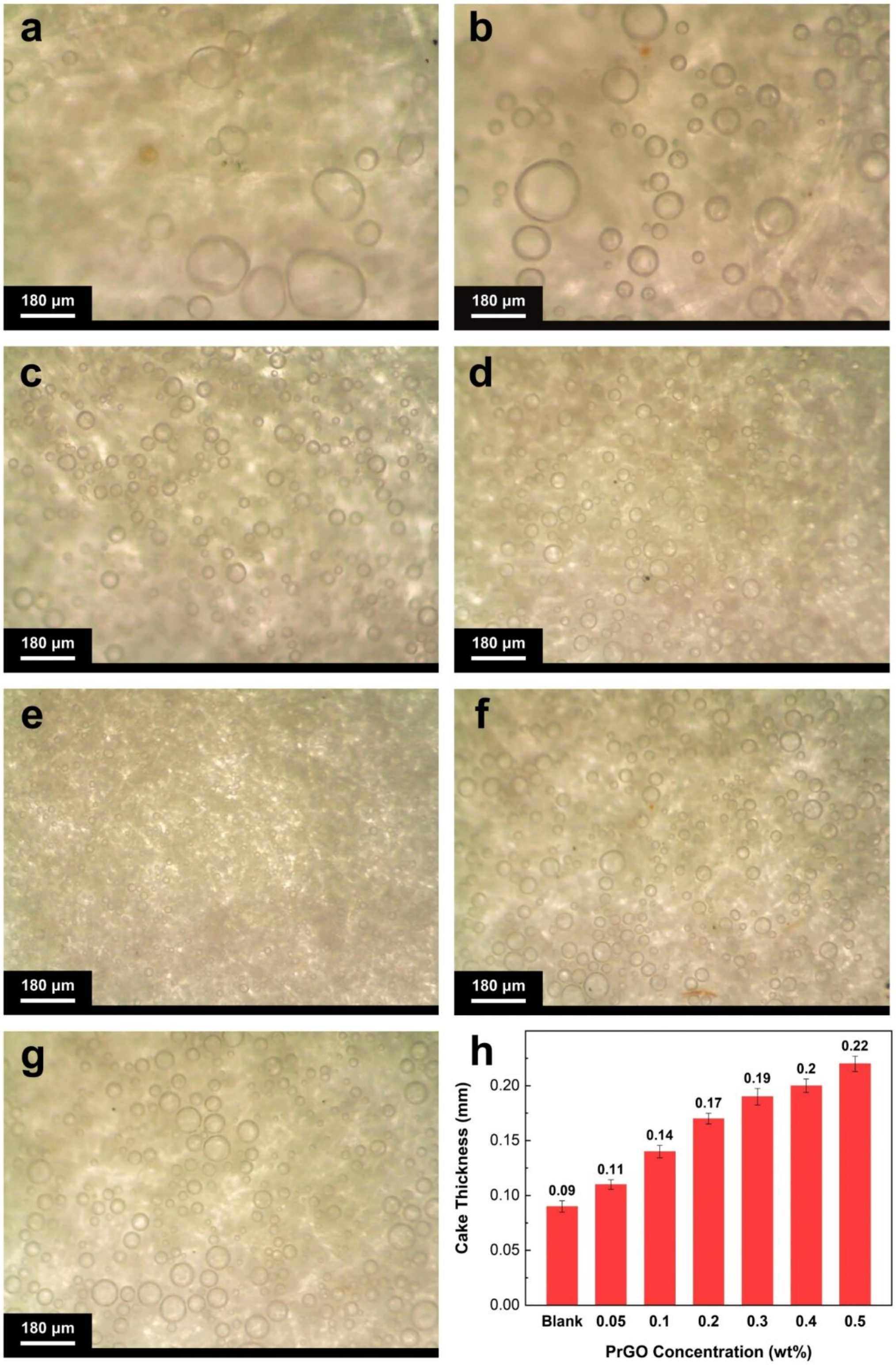

Figure S2. Optical micrographs of aphronic fluid filtration cake. The effect of PrGO concentrations: **a**) blank, **b**) 0.05 wt%, **c**) 0.10 wt%, **d**) 0.20 wt%, **e**) 0.30 wt%, **f**) 0.40 wt%, and **g**) 0.50 wt%. **h**) cake thickness.

Table S1. Concentrations of the used materials in the formulation of the AMB water-based drilling fluid

Materials	Concentration (wt%)	Mixing time (min)	Function
Deionized water	90.00	-	Base fluid as a continuous phase
Sodium Carbonate	0.10	15	Hardness Buffer
Sodium Hydroxide	0.06	5	pH Controller
Xanthan Gum	0.50	20	Viscosifier
PAC-LV	0.80	15	filtration controller
Modified Starch	1.00	15	Fluid loss Controller
Biocide	0.04	2	Bacteria Controller
Mixed Surfactant	7.50	4	Aphronizer

Table S2. Low shear rate viscosity (LSRV) of the graphene-loaded AMB water-based drilling fluids at different concentration of PrGO. The high LSRV is an important criterion for the microbubbles to be effective in bridging and pore-blocking ability.

		PrGO concentration (wt%)					
	**Blank**	**0.05**	**0.10**	**0.20**	**0.30**	**0.40**	**0.50**
**LSRV (cP) at 0.6 rpm**	54,000	87,000	104,000	123,000	139,000	121,000	94,000
**LSRV (cP) at 0.3 rpm**	125,000	159,000	178,000	202,000	224,000	197,000	166,000
